# Metabolic profiles in drought-tolerant wheat with enhanced abscisic acid sensitivity

**DOI:** 10.1371/journal.pone.0307393

**Published:** 2024-07-22

**Authors:** Yuanjie Weng, Ryosuke Mega, Fumitaka Abe, Hisashi Tsujimoto, Masanori Okamoto

**Affiliations:** 1 United Graduate School of Agricultural Science, Tokyo University of Agriculture and Technology, Tokyo, Japan; 2 Center for Bioscience Research and Education, Utsunomiya University, Utsunomiya, Japan; 3 RIKEN Center for Sustainable Resource Science, Yokohama, Japan; 4 Graduate School of Sciences and Technology for Innovation, Yamaguchi University, Yamaguchi, Japan; 5 Division of Basic Research, Institute of Crop Science, National Agriculture and Food Research Organization (NARO), Tsukuba, Japan; 6 Arid Land Research Center, Tottori University, Tottori, Japan; 7 Kihara Institute for Biological Research, Yokohama City University, Yokohama, Kanagawa, Japan; Louisiana State University College of Agriculture, UNITED STATES OF AMERICA

## Abstract

Global warming has led to the expansion of arid lands and more frequent droughts, which are the largest cause of global food production losses. In our previous study, we developed TaPYLox wheat overexpressing the plant hormone abscisic acid (ABA) receptor, which is important for the drought stress response in plants. TaPYLox showed resistance to drought stress and acquired water-saving traits that enable efficient grain production with less water use. In this study, we used TaPYLox to identify ABA-dependent and -independent metabolites in response to drought stress. We compared the variation of metabolites in wheat under well-watered, ABA treatment, and drought stress conditions using the ABA-sensitive TaPYLox line and control lines. The results showed that tagatose and L-serine were ABA-dependently regulated metabolites, because their stress-induced accumulation was increased by ABA treatment in TaPYLox. In contrast, L-valine, L-leucine, and DL-isoleucine, which are classified as branched chain amino acids, were not increased by ABA treatment in TaPYLox, suggesting that they are metabolites regulated in an ABA-independent manner. Interestingly, the accumulation of L-valine, L-leucine, and DL-isoleucine was suppressed in drought-tolerant TaPYLox under drought stress, suggesting that drought-tolerant wheat might be low in these amino acids. 3-dehydroshikimic acid and α-ketoglutaric acid were decreased by drought stress in an ABA-independent manner. In this study, we have succeeded in identifying metabolites that are regulated by drought stress in an ABA-dependent and -independent manner. The findings of this study should be useful for future breeding of drought-tolerant wheat.

## Introduction

The world population is projected to increase to about 9 billion by 2050 [1]. Particularly in Africa, about 870 million people are already chronically undernourished, and solving this problem will require a 60%–110% increase in global agricultural production [2]. Drought stress in crop cultivation is the most important environmental constraint for sustainable agriculture and the leading factor in reducing global food productivity [3, 4]. Wheat (*Triticum aestivum* L.), one of the three major cereal grains, is a major source of calories for about 4.5 billion people living in developing countries [5]. However, wheat yields are estimated to decrease by about 6% for each 1°C increase in global mean temperature, in addition to losses caused by frequent exposure to drought exacerbated by climate change [6, 7]. For example, during the so-called Millennium Drought (2002–2009), Australian wheat yields were up to 25% lower than average. Therefore, breeding drought-tolerant wheat cultivars with high water-use efficiency is a high priority for maintaining a sustainable food supply. Therefore, it is necessary to understand in detail the physiological and metabolic responses of wheat to drought stress.

Many signaling molecules, such as abscisic acid (ABA), intracellular calcium ions (Ca^2+^), and reactive oxygen species (ROS), play important roles in the drought stress response in plants [8]. ABA is central to the drought stress response of plants, and a core set of ABA metabolism and signaling genes was revealed using the model plant *Arabidopsis thaliana* [9]. When plants are exposed to drought stress, ABA biosynthesis is induced, then the increased ABA binds to receptors and triggers signals transduction, leading to cellular responses, such as stomatal closure [10]. When endogenous ABA levels rise in response to water deficiency, this leads to the formation of a ligand–receptor complex that stimulates the activity of clade A protein phosphatase 2C (PP2C), a negative regulator of ABA signaling [11–13]. As a result, the protein phosphorylation activity of SnRK2 kinase, which is normally suppressed by PP2C, is restored, and target proteins required for stomatal closure, such as anion channels and the bZIP transcription factor, are phosphorylated. Phosphorylation activates target proteins and exerts adaptation and defense mechanisms against drought stress [10]. Drought stress-responsive genes are divided into two types, namely those showing either ABA-dependent or ABA-independent regulation by drought stress [14]. *Cis*-element analysis revealed a conserved ABA-responsive element (ABRE) with an ACGT core in the promoter regions of ABA-dependent drought stress-responsive genes [15].

ABRE-binding proteins (AREB) or ABRE-binding factors (ABF), which are basic-domain leucine zipper (bZIP) transcription factors, are primarily responsible for regulating the expression of these genes [16]. Among the *Arabidopsis* AREB/ABF subfamily, AREB1/ABF2, AREB2/ABF4 and ABF3 are induced by both drought stress and ABA [17]. However, several genes that are responsive to drought stress do not respond to ABA, indicating that there might be an ABA-independent regulation mechanism for drought stress [14]. Drought stress-responsive gene promoters are ABA-independent and contain *cis*-elements such the DRE (drought-responsive element) and CRT (*cis*-acting element). Gene expression is regulated by transcription factors such as CBF. Transcription factors such as these play crucial roles in ABA-dependent gene expression under drought stress [14, 18, 19]. In addition, it has been shown that regulation of the drought stress response in plants involves ABA-independent transcription factors, including MYB/MYC and WRKY [20].

Metabolomics provides a useful analytical tool to analyze biological and physiological variations brought on by environmental and gene expression changes [6]. Metabolites are at the end of a multi-level cascade of gene and protein function and are therefore useful tools for analyzing cellular stress responses in plants [21]. In addition, the same metabolites occur widely among plant species, unlike some gene sequences and proteins. Species-specific metabolic changes can be captured, as well as metabolic changes common to multiple species. Recently, metabolomics analyses have revealed changes in primary and secondary metabolites that are associated with drought responses in several plant species. In *Arabidopsis*, most amino acids (including proline, glutamine, tryptophan, alanine, aspartic acid, ornithine, isoleucine, leucine, valine), tricarboxylic acid cycle (TCA) intermediates (including 2-oxoglutarate, cis-aconitic acid, succinate), flavonoids (including quercetin, cyanidin) and lipids (including glycosyl inositol phosphoceramides, acylated steryl glycosides) were increased by drought stress [22, 23]. In wheat, proline, tryptophan, organic acids, phenols, and sulfur-related metabolites (glutathione, methionine, cysteine) have been reported to accumulate under drought stress [24, 25].

The drought stress response in wheat has been widely studied at the genetic level and in metabolomics research. Metabolomic analyses in response to salt stress [26], temperature [27], nitrogen nutrition [28], and drought stress [24] have been reported. Previous study has shown that wheat TaPYLox, which is overexpressed through ABA receptors, exhibits high sensitivity to ABA, drought tolerance, and water-saving properties [29]. In this study, we used TaPYLox to identify metabolites and gene expression in response to drought stress in both ABA-dependent and -independent manners. Using TaPYLox as a model, we also searched for metabolite markers that characterize the drought-tolerant trait in wheat.

## Materials and methods

### Plant materials

Bread wheat (*Triticum aestivum* L.) used was cv. Fielder. The TaPYL4 overexpressing strain (TaPYLox) was a transgenic wheat strain generated with the wheat ABA receptor TaPYL4 in the Fielder background using the maize ubiquitin promoter, as published in [29] (hereafter referred to as lines 8–5 and 17–2). A segregating line (hereafter referred to as Null), generated in the process of creating TaPYLox but which did not contain the transgene, was used as a control line.

### Cultivation methods and growth conditions

Wheat seeds were sterilized for 10 min with 5% sodium hypochlorite, then rinsed three times with sterile water. Sterilized seeds were placed in a Petri dish containing filter paper wetted with sterile water. Seeds were cold treated for 2 d at 4°C in the dark then incubated for 5 d in growth chambers at 18–22°C, 65%–75% humidity and 16-h daylength to germinate. Conditions were monitored using an artificial meteorological instrument set LH-411PFD-S (S) (Japanese Medical Instrument Mfg. Co., Ltd.). Seedlings were raised in soil (Cainz). Four individuals were transplanted into one pot (height 100 mm, top diameter 120 mm, bottom diameter 80 mm, capacity 0.6 L) and cultivated at 20°C for 14 h with light intensity c. 4400 lx, nighttime temperature 15°C for 10 h, and 60%–90% humidity. In the drought stress treatment over time, Null plants were divided into three pots at the wheat jointing stage, one month after transplanting, each for well-watered and drought stress treatments. The total weights of pots and plants were recorded on days 0, 2, 4 and 6 during the drought stress treatment. On the second day without watering, the moisture content of the planted soil decreased dramatically. As the drying treatment progressed, the loss of moisture content continued. Loss of soil moisture increased to 34% on day 2 (DCd2) and from 43% to 46% on DCd4 and DCd6, indicating progressively increasing drought stress in the plants (S1a Fig). On the second day of the drying treatment, the leaves of the plants showed slight wilting, but from the fourth day onwards, the wilted state of the plants intensified, and on the sixth day the plants showed severe wilting (S1b Fig). To understand how plant temperature changed with different levels of drought stress, we conducted thermal analysis of plants under drought stress over time. On DCd2, the temperature of the drought-stressed plants began to rise (S1b Fig). As the drying intensified (DCd4), the temperature also increased. In DCd6, a rapid rise in temperature was observed as a result of excessive water deficit (S1b Fig).

Null, 8–5 and 17–2 plants were divided into three pots at the wheat jointing stage, one month after transplanting, each for well-watered, ABA and drought stress treatments. ABA-treated plants were well-watered but were given 10 μM ABA solution from the bottom of the tray and 25 μM ABA solution was sprayed on the aerial parts of the plant. In the drought stress treatment, no water was supplied after the start of the treatment. Null and 8–5 plants were weighed on days 0, 2, 3, and 5 under drought stress conditions.

### Thermo-imaging analysis

Plant surface temperatures were monitored with an RS00SR-S Infrared imaging camera (Nippon Avionics Co., Ltd.). The acquired thermal images for each plant were analyzed using an InfRec Analyzer NS9500 Standard (Nippon Avionics). Photos of plants were taken with an EM-5 mark II digital camera (Olympus).

### Extraction and derivatization for metabolomics analysis

For each treatment and condition, 16 wheat leaves were collected in a 50-mL Falcon tube. After freezing in liquid nitrogen, the samples were freeze-dried for more than 12 h (VD-25OR freeze dryer, TAITEC). The freeze-dried samples were crushed (Shakemaster BMS-A20TP, 1000 rpm, 10 min), then divided into 10-mg portions in two 3-mm stainless steel 2-mL tubes and crushed again (MM301, Retsch, speed 16 Hz, 5 min). Mix solvent (MeOH/H_2_O/CHCL_3_ = 5:2:2) (1000 μL) and 20 μL of 0.2 mg/mL ribitol solution were added. The samples were mixed with a vortex mixer for 5 min, shaken at 37°C for 30 min, then centrifuged at 4°C and 8,000 rpm for 10 min. After centrifugation, 900 μL of the supernatant was mixed with 400 μL sterile water, then centrifuged at 4°C, 16,000 rpm, for 5 min. After centrifugation, 400 μL of the supernatant was transferred to a new 1.5-mL tube, then concentrated with a centrifugal concentrator (Thermo Fisher Scientific SpeedVac SPD121P) at room temperature. Samples were removed from the concentrator, frozen in liquid nitrogen, and freeze-dried overnight (VD-25OR freeze dryer, TAITEC). Methoxyamine hydrochloride (35 μL of 20 mg/mL in pyridine) was added to the samples, mixed with a vortex mixer for 5 min and then shaken at 30°C for 90 min. Then, 25 μL MSTFA was added and mixed with a vortex mixer for 5 min, followed by shaking for 90 min at 30°C. After centrifugation at 22°C and 13 000 rpm for 5 min, 50 μL of the supernatant was taken and transferred to a vial for GC–MS and stored at −20°C until analysis.

### Metabolite analysis

Samples were analyzed using Agilent Technologies 7890B GC–MS system with MS ion source 230°C, MS quadrupole 150°C, high vacuum 2.40e−06 Torr. Samples were injected in a 1:20 split ratio in a special block. Separation was achieved using a 40 m DB-5ms column with a film thickness of 0.25 μm (Agilent), and 0.9 mL per min helium gas flow rate. Retention time for myristic acid-d27 was locked to 16.727 min. The program consisted of 60°C for 1 min, a ramp of 10°C per min to 325°C, and 10-min hold. Other specifications included transfer line temperature held at 330°C, MS using electron ionization mode (EMV tuning 1460), atune.u (Autotune, tuned for maximum response over the full scan range), and solvent waiting time of 5.9 min. Mass-to-charge ratios in the range 50–650 m/z were scanned at 2.5 scans per sec after electron impact ionization.

Data files from GC–MS experiments were converted to.cef format and processed using Unknowns Analysis (Agilent Technologies Inc.) to create a matrix of molecular features as defined by retention index and mass/charge ratios (m/z). Upon collection of retention times for metabolites (ranging from C8 to C30) using myristic acid-d27 (Agilent Fiehn GC/MS Metabolomics Standards Kit, USA) as the standard, the Agilent Fiehn Metabolome Database was used to obtain the corresponding retention index.

### Real-time PCR

For RNA extraction, approximately 50 mg of crushed sample was dispensed while freezing in liquid nitrogen into a 2-mL tube containing 3-mm and 5-mm stainless steel beads, and crushed to a fine powder. Frozen samples were thawed on ice and RNA was extracted using the Plant Total RNA purification kit (BioElegen Technology). Total RNA (500 ng) was reverse-transcribed using RevTra Ace qPCR RT Master Mix with gDNA Remover (TOYOBO) according to the manufacturer’s protocol. QRT-PCR was performed using the LightCycler^®^ 480 System (Roche), KOD SYBR qPCR Mix (TOYOBO) and gene-specific primer sets shown in S1 Table. For normalization of data, the *TaActin* gene was used as an internal standard.

### Statistical analysis

Metabolite contents were compared using one-way ANOVA for both lines and cultivars, with P-value ≤ 0.05, fold change 1.25, and Z-transformation. Benjamini–Hochberg correction was systematically applied across all t-tests and ANOVA metabolomics results to account for falsely rejected statistical hypotheses when conducting multiple comparisons, termed false discovery rate (FDR). Principal component analysis (PCA) and hierarchical cluster analysis were conducted using the Mass Profiler Professional software (Agilent Technologies, USA).

## Results

### Metabolome analysis using GC–MS under drought stress over time

Metabolites detected by GC–MS were analyzed by Unknowns Analysis and Mass Profiler Professional. In the drought stress over time experiment, in total, around 340 compounds were detected in Null, and around 130 were identified (S2a Fig and S2 Table). By hierarchical cluster analysis (S2b Fig and S3 Table), the control sample WWd0 was clearly separated from all drought stress-treated samples. DCd2, WWd4, and WWd6 were clustered together. In contrast, when the stress was more intense with longer exposure to drought (DCd4 and DCd6), the samples were clustered together, suggesting a significant change in metabolism (S2b Fig). Principal component analysis (PCA) was performed to visualize the relationships among the samples (S2c Fig). The contribution of the first principal component (PC1) was 27.89%, and the contribution of the second principal component (PC2) was 9.18%. Changes over time when plants were exposed to stress conditions were well separated by PC1. Well-watered samples (WWd0, 2, 4, and 6) all tended to cluster, indicating that they had similar metabolite profiles. Samples grown under moderate drought conditions (DCd2) clustered in the direction of the well-watered samples. Samples of DCd4 were located in the intermediate region between moderate and severe drought (S2c Fig), suggesting that DCd4 was in a transition state. Because DCd6 was well separated from the area of the wet treatments, we surmised that the compound group changed depending on the extent of drought stress (S2c Fig). Venn diagrams were used to illustrate the patterns of changes in metabolites over time (S3 Fig). Overlap analysis showed that eight metabolites (L-tryptophan, L-valine, L-leucine, glycerol 1-phosphate, L-threonine, gluconic acid lactone, malonic acid, D-malic acid) were increased and two metabolites (quinic acid, aspartic acid) were decreased at all time points. These metabolites probably responded most clearly to drought stress and might prove useful as biomarkers (S3 Fig and S4 Table).

### Drought stress treatment in TaPYLox

**Fig 1:**
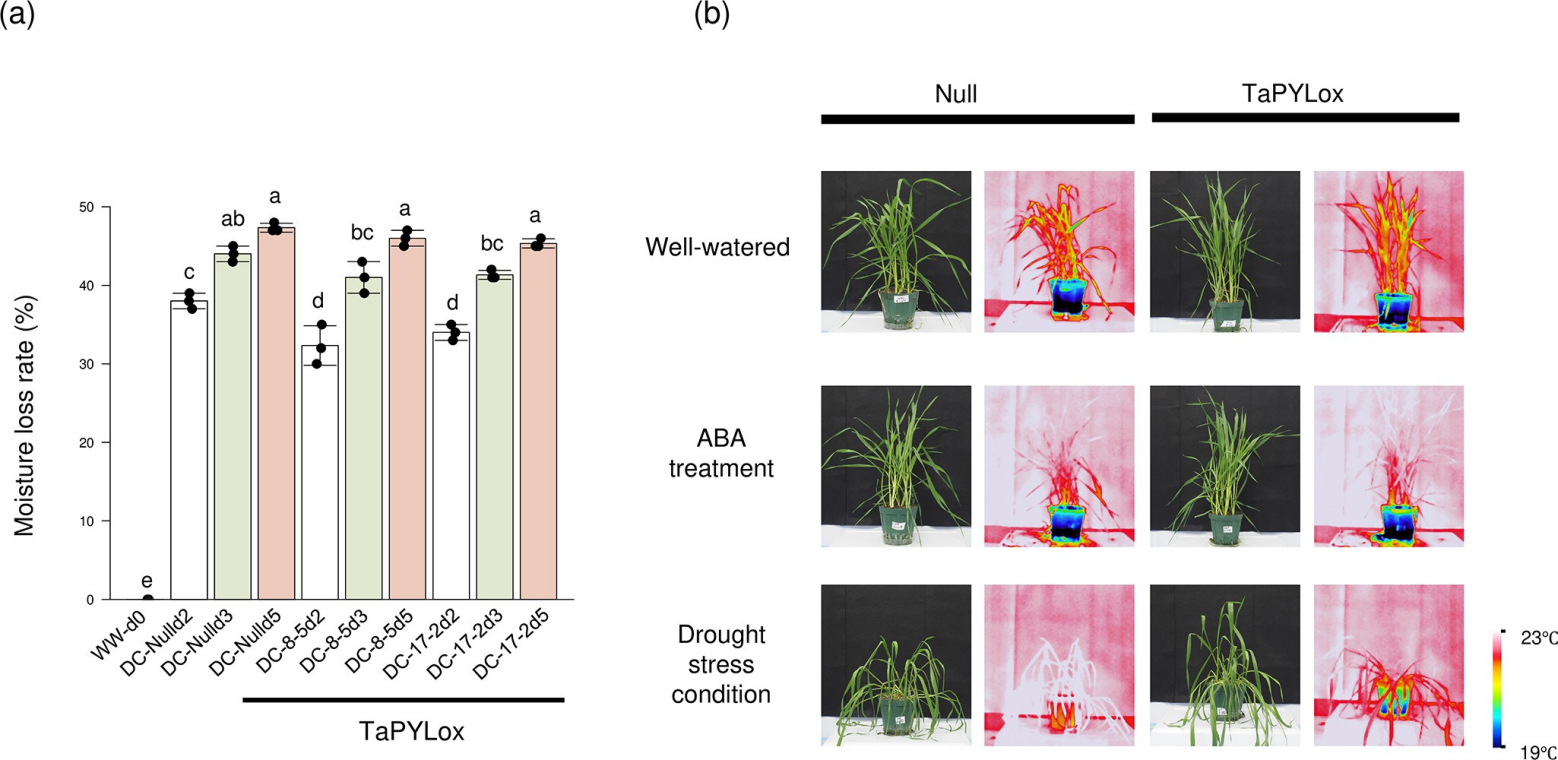
Morphological and temperature changes in TaPYLox wheat under drought stress treatment. (a) For drought condition (DC), water supply was stopped 31 d after transplanting, and water loss (%) was measured at days 0, 2, 3, and 5. WW (Well-Watered) indicates watered control plants. Results are shown as mean and standard deviation with three replications. Different letters indicate significant differences (Tukey–Kramer test, P < 0.05). (b) Abscisic acid (ABA) was applied from 31 d after transplanting, and drought condition was applied at the same time. Plant morphology photographs and thermal images are shown for the control line (Null) and TaPYLox under well-watered, ABA treatment and drought stress conditions after 5 d.

To assess the level of drought stress, we measured the soil moisture content during plant cultivation. Moisture loss was observed on days 2 and 3 of drought condition (DC treatment), and this loss was slightly reduced in TaPYLox. On the 5th day of the drought treatment (DCd5), both Null and TaPYLox were thought to have been treated with substantially the same amount of drought stress, so samples were collected for chemical analysis at that time (Fig 1a). No significant differences between Null and TaPYLox were observed in the well-watered (WW) and ABA treatments (Fig 1b). In contrast, under the drought treatment, Null showed a significantly wilted state, while TaPYLox also showed wilting, but to a milder degree than Null (Fig 1b). Thermal analysis of the plants was performed to monitor the changes in temperature of the plants under different conditions. With ABA treatment, plants showed a slight increase in temperature over well-watered (WW). Null and TaPYLox were both observed to have a rapid increase in plant temperature under drought conditions. Water was lost from Null’s pots through transpiration, resulting in higher leaf temperatures. However, because TaPYLox suppresses transpiration, less water was lost, so the water remained in the pot for a longer period of time, and the leaf temperature was kept lower than that of Null (Fig 1b).

### Metabolome analysis using GC–MS under drought stress in TaPYLox

**Fig 2:**
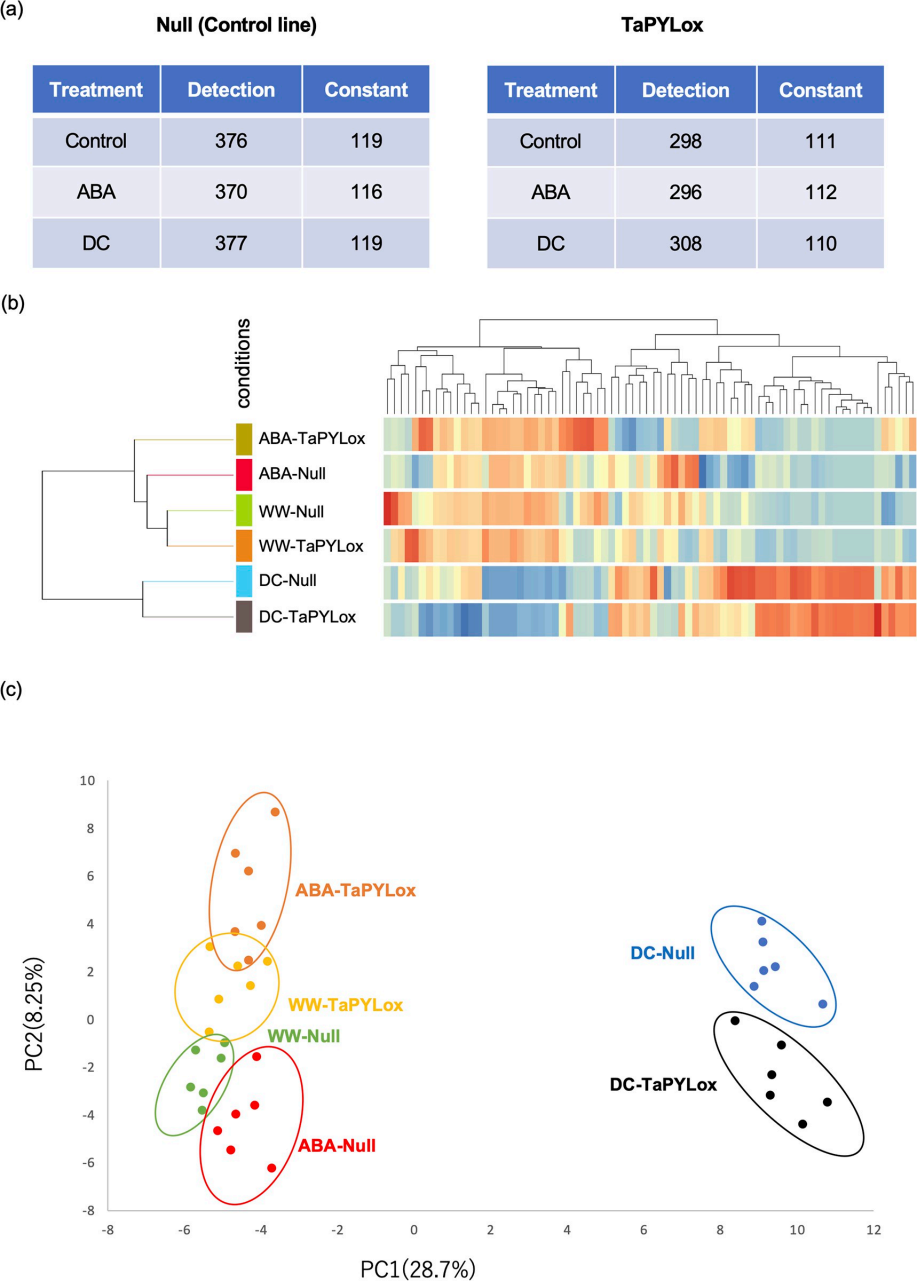
Metabolites identified by GC–MS. (a) Data from GC–MS experiments were processed by Unknowns Analysis (Agilent Technologies) to identify metabolite quantities. The number of replications was six. The values in the table are calculated from the average of 6 replications. Detection denotes the total number of metabolites detected; Constant denotes the number of metabolites identified in common across different treatments. (b) After statistical analysis (one-way ANOVA, P-value ≤ 0.05, fold change 1.25), a hierarchical cluster tree was created using MPP (Mass Profiler Professional) to collect and visualize compounds with similar patterns of variation. The red color indicates high accumulation, yellow an intermediate accumulation, and blue a low accumulation. (c) Principal component analysis was used to examine the interrelated effects of WW, ABA and DC on control (Null) and drought resistant (TaPYLox) lines. After statistical analysis (one-way ANOVA, P-value ≤ 0.05, fold change 1.25), the similarity of each sample group was analyzed by setting the principal components to two dimensions.

To investigate the reproducibility of biological replicates and the relationship between different samples (genotype, stress treatment), we performed correlation analysis based on global metabolite accumulation patterns. The number of compounds detected in the control plants and TaPYLox was around 380, of which we were able to identify around 116 (Fig 2 and S5 Table). All seven samples were classified into three groups. Compounds showing high reproducibility between biological replicates are shown by a heatmap (Fig 2b and S6 Table). ABA treatment of Null and TaPYLox showed similar accumulation patterns to well-watered conditions, indicating that ABA-induced metabolite changes were mild. In contrast, under drought stress treatment, both Null and TaPYLox showed large changes in metabolites compared with the well-watered condition and ABA treatment. In TaPYLox, some of the compounds that increased with ABA treatment also increased with drought stress treatment, suggesting that a group of ABA-dependent drought-induced substances might be identifiable (Fig 2b). Principal component analysis (PCA) was performed to reduce the dimensionality of the data and visualize the relationships between samples (Fig 2c). The contribution of the first principal component (PC1) was 28.7%, and the contribution of the second principal component (PC2) was 8.25%. The different genotypes were clearly separated by PC2, and the time the plants were exposed to stress conditions was well separated by PC1. With ABA treatment, PC1 was less affected, which we consider to be the effect of metabolites mainly determined by PC2. Also, the drought stress treatments were clustered and well separated from the well-watered condition and the ABA treatment, indicating that the metabolites affected by drought were very different. Null and TaPYLox were plotted close together in WW, ABA and DC, suggesting that the metabolites of Null and TaPYLox changed only slightly (Fig 2c).

### Metabolic changes with ABA or drought stress treatment in Null and TaPYLox

We compared the changes in metabolite contents under ABA and drought treatments between Null and TaPYLox genotypes. In Null, five metabolites showed increased content with ABA treatment and 30 metabolites under drought stress, with three overlapping metabolites. In contrast, 16 metabolites were reduced by ABA treatment in Null, and 19 metabolites by drought stress, with five overlapping metabolites (S4a Fig and S7 Table). Similarly, TaPYLox showed an increase in 13 metabolites following ABA treatment and 33 metabolites following drought stress, with seven overlapping metabolites. Seven metabolites were reduced by ABA treatment in TaPYLox, and 20 metabolites by drought stress, with four overlapping metabolites (S4b Fig and S7 Table). Comparison between Null and TaPYLox revealed that 25 metabolites increased in TaPYLox under drought stress treatment while 12 metabolites decreased (S4c Fig and S7 Table).

**Fig 3:**
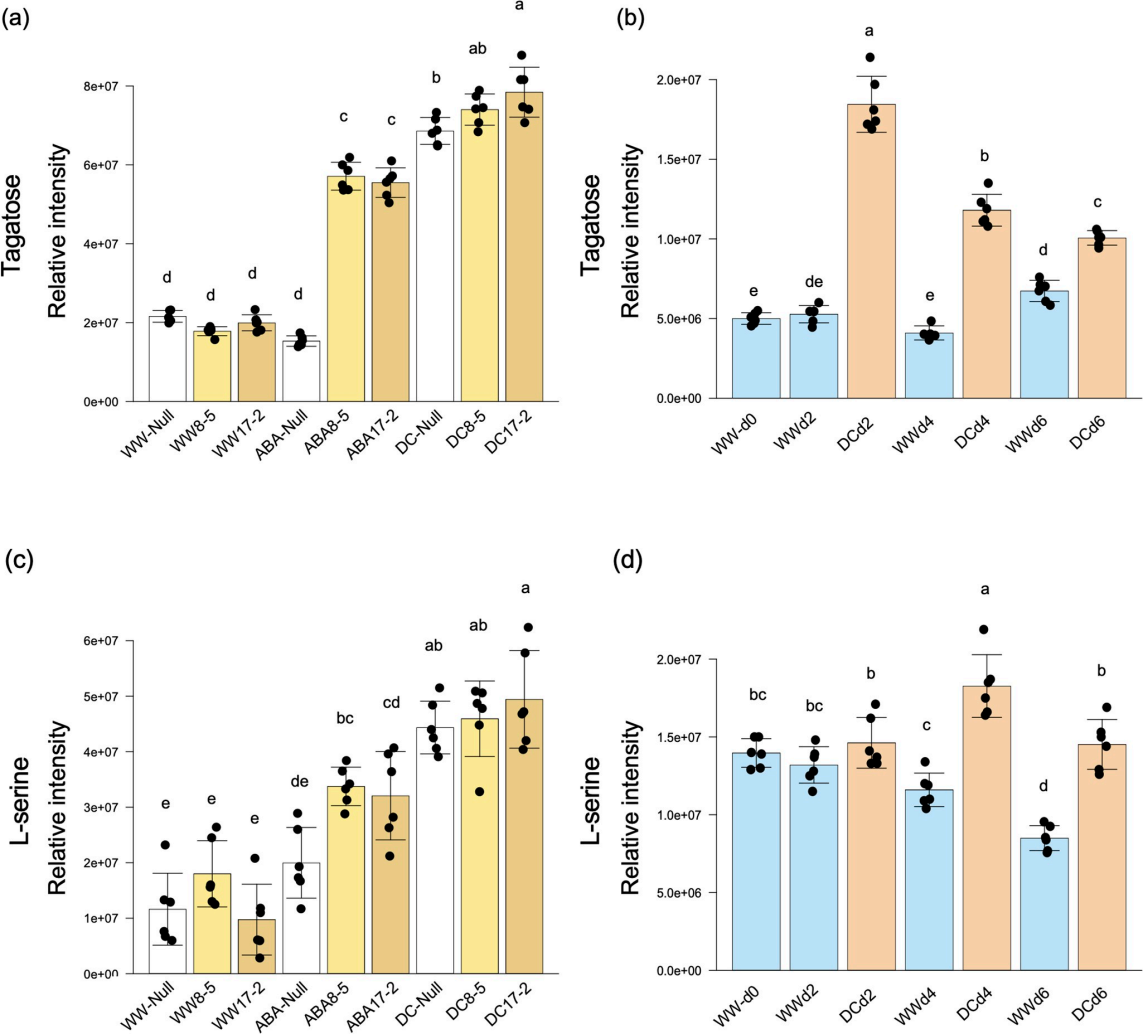
Metabolites that accumulate with ABA or drought treatment. (a) and (c) content of tagatose (a) and L-serine (c) in control (Null) and TaPYLox lines (8–5 and 17–2) under well-watered condition (WW), ABA treatment (ABA) and drought condition (DC). (b) and (d) content of tagatose (b) and L-serine (d) in Null during drought stress treatment over time (days 0, 2, 4, 6). Mean and standard deviation with six replications. Different letters indicate significant differences (Tukey–Kramer test, P < 0.05).

**Fig 4:**
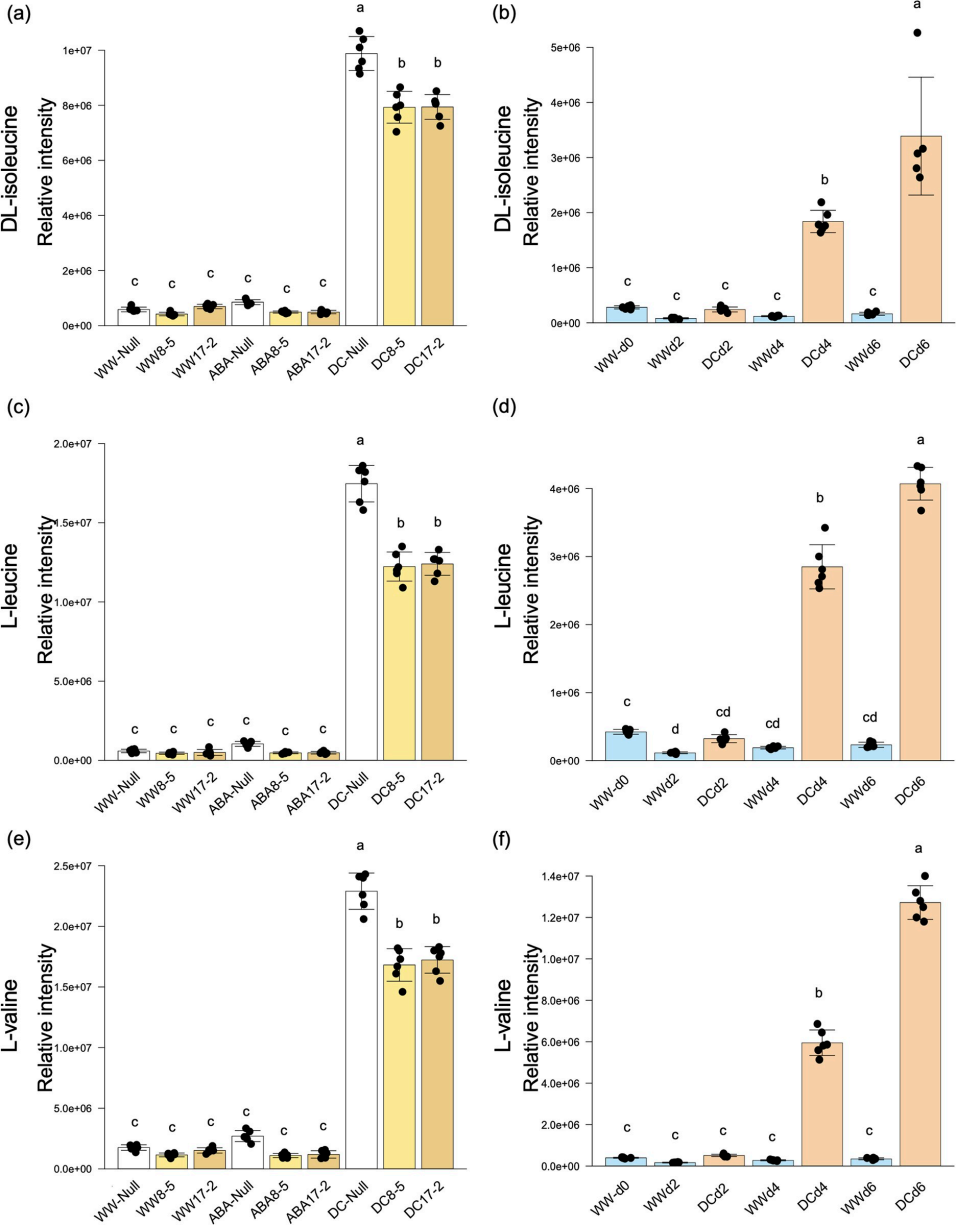
Metabolites that accumulate under drought stress in an ABA-independent manner. (a), (c) and (e) content of DL-isoleucine, L-leucine and L-valine in control (Null) and TaPYLox lines (8–5 and 17–2) under well-watered condition (WW), ABA treatment (ABA) and drought condition (DC). (b), (d) and (f) metabolite variation in Null under drought stress treatment over time (days 0, 2, 4, 6). Mean and standard deviation with six replications. Different letters indicate significant differences (Tukey–Kramer test, P < 0.05).

**Fig 5:**
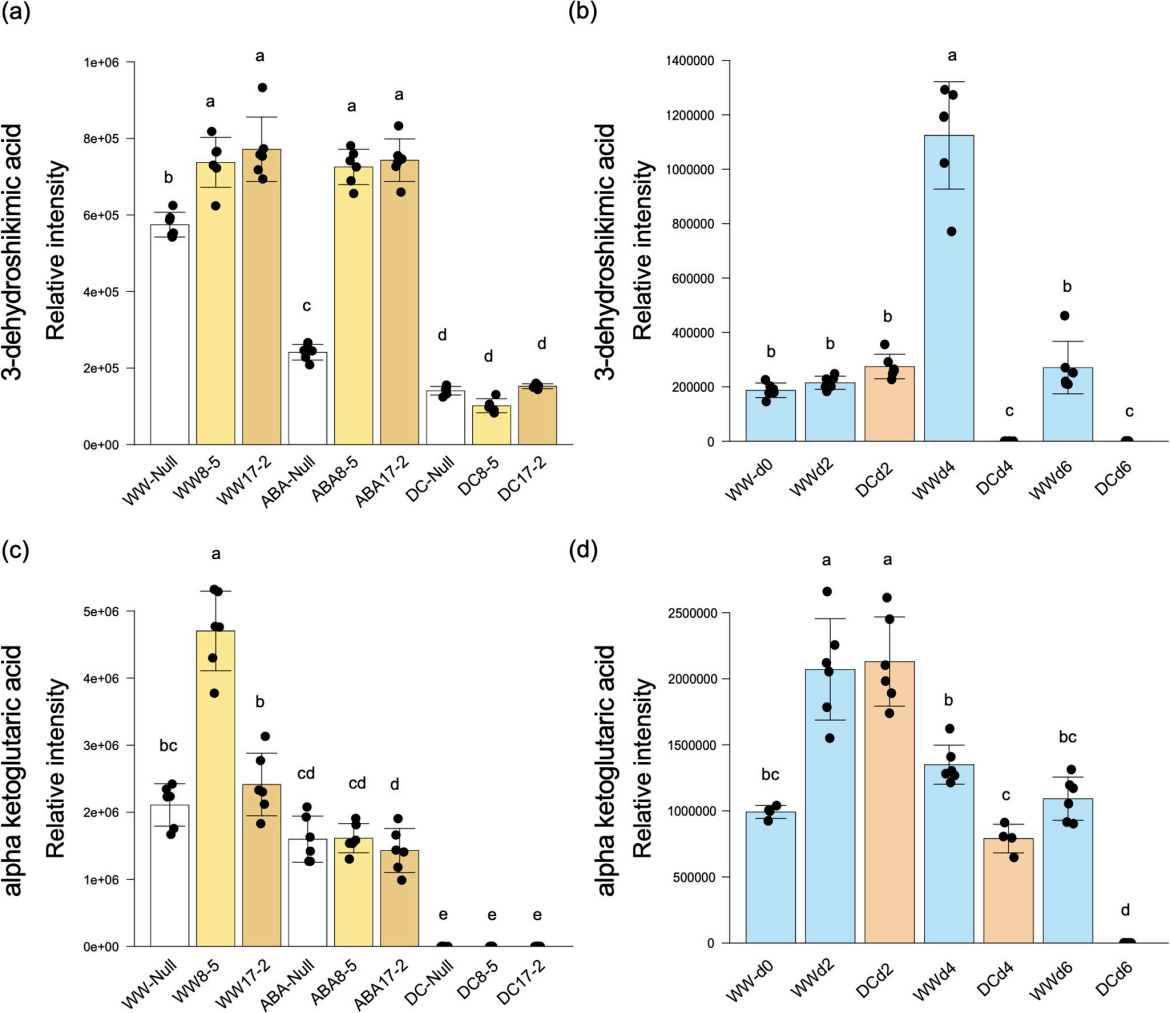
Metabolites reduced by drought stress. (a) and (c) content of 3-dehydroshikimic acid and α-ketoglutaric acid in control (Null) and TaPYLox lines (8–5 and 17–2) under well-watered condition (WW), ABA treatment (ABA) and drought condition (DC). (b) and (d) metabolite content in Null during drought stress treatment over time (days 0, 2, 4, 6). Mean and standard deviation with six replications. Different letters indicate significant differences (Tukey–Kramer test, P < 0.05).

Tagatose and L-serine accumulated in ABA-treated TaPYLox, and accumulated in both Null and TaPYLox under drought stress conditions (Fig 3a and 3c and S6 Table). Null also accumulated tagatose and L-serine in drought stress treatment over time (Fig 3b and 3d and S3 Table). This result suggests that tagatose and L-serine increase in a way that is related to ABA. In contrast, L-valine, L-leucine and DL-isoleucine, which are branched chain amino acids (BCAAs), accumulated in both Null and TaPYLox genotypes under drought stress conditions but not with ABA treatment (Fig 4a, 4c and 4e and S6 Table). Correspondingly, in the drought stress treatment over time, the content of BCAAs in Null increased over time, with the greatest accumulation in DCd6 when the degree of drought stress was particularly intense (Fig 4b, 4d and 4f and S3 Table). In other words, BCAAs are considered to be compounds that increased with drought stress in an ABA-independent manner. However, the accumulation of these metabolites was less in TaPYLox than in Null (Fig 4a, 4c and 4e and S6 Table). 3-dehydroshikimic acid and α-ketoglutaric acid are also considered to be compounds that are reduced by drought stress in an ABA-independent manner (Fig 5, S3 and S6 Tables).

### Gene expression analysis of drought stress response

We analyzed the gene expression of metabolic enzymes related to primary metabolites. The expression level of the L-serine inactivating gene *SR1* was reduced in ABA-treated TaPYLox, and further reduced by drought stress treatment in both Null and TaPYLox lines (S5a and S5b Fig). This result was consistent with the finding that L-serine increased with ABA treatment (Fig 3 and S6 Table). TaBCAT (Branched-Chain amino acid Transaminase) is an enzyme involved in the metabolism of branched-chain amino acids, playing a crucial role in their metabolic processes. The involvement of BCATs in stress responses has been reported in Arabidopsis [30, 31], and other plant species [32, 33]. *TaBCAT* increased during ABA treatment and the early stages of drought stress, but there was no correlation between its gene expression and the accumulation of BCAAs (Fig 4, S6a and S6b Fig). The biosynthetic enzyme gene *EMB3004*, which is related to 3-dehydroshikimic acid metabolism, showed downregulation in both Null and one of the TaPYLox lines by drought stress treatment (S6c and S6d Fig). This gene expression was consistent with variations in 3-dehydroshikimic acid (Fig 5a).

Late embryogenesis abundant (LEA) proteins are hydrophilic proteins that accumulate during seed formation when seeds are dehydrated, and in plants they protect cells against desiccation and cold stress [34]. Because the LEA protein is closely associated with the acquisition of drought resistance, we also analyzed the genes related to LEA protein biosynthesis. Gene expression levels were dramatically increased by drought stress in both Null and TaPYLox lines under drought stress (S7a and S7b Fig). It is well known that the *LEA* gene responds to ABA and drought stress. Although the induction by ABA was low, the expression level of the *LEA* gene was higher in TaPYLox than in Null under ABA treatment and drought stress treatment. Therefore, we surmise that ABA and drought stress acted synergistically to increase the expression of the *LEA* gene.

## Discussion

Drought is a major environmental stressor affecting plant growth. Therefore, elucidating the mechanisms of drought stress tolerance in wheat is important to develop strategies for drought-tolerant wheat breeding. Metabolomic analysis has become established as an important analytical tool for understanding biochemical processes. In this study, we used metabolomic analysis to investigate the ABA-dependent and -independent drought stress responses in wheat by characterizing two wheat lines, a control line (Null) and a drought-tolerant TaPYLox with high ABA sensitivity.

While ABA-dependent pathways are pivotal, plants also possess ABA-independent mechanisms to cope with drought stress. The most significant changes occurred for amino acids, organic acids, and sugars. Proline and BCAAs, which are known to protect plants against abiotic stress, were more abundant in the drought treatments. Similarly, increased levels of amino acids were recorded under drought stress in other wheat studies [35].

**Fig 6:**
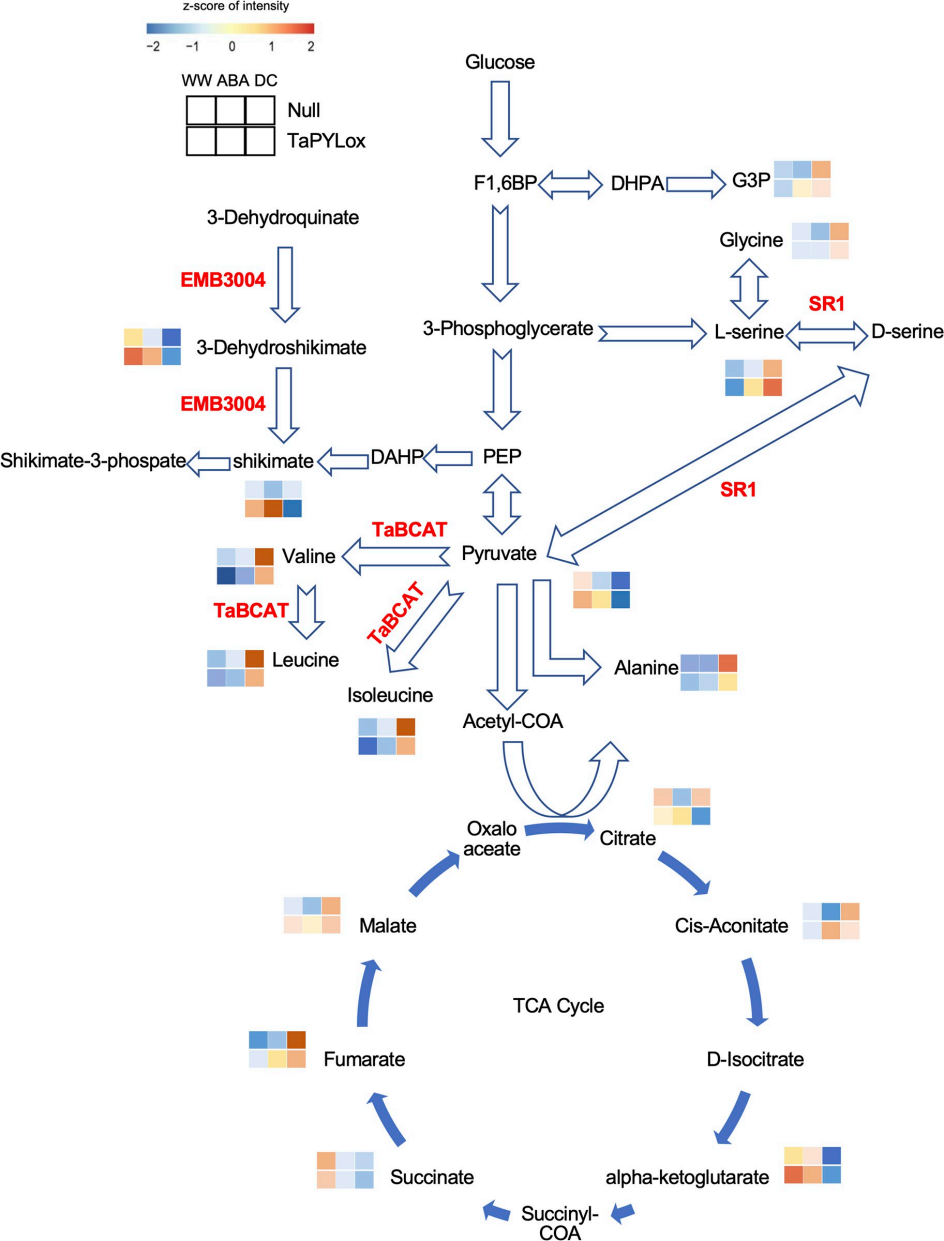
Comparison of control line (Null) and TaPYLox in primary metabolic pathways. Changes in metabolites involved in the amino acid pathway and citric acid cycle in Null and TaPYLox are shown by color coding of z-scores (top row Null, bottom row TaPYLox) under well-watered (WW), ABA or drought (DC conditions). P-value ≤ 0.05, fold change 1.25.

The BCAAs, L-valine, L-leucine and DL-isoleucine, accumulated in both Null and TaPYLox lines under drought stress conditions (Fig 4a, 4c and 4e). The accumulation of BCAAs increased remarkably with the progression of drought stress from DCd4. Previous studies have suggested that BCAAs provide an alternative energy source in sugar-starved *Arabidopsis* [36] and drought-stressed wheat [24]. BCAAs have also been shown to function as alternative electron donors in the respiratory system during abiotic stress [37]. Furthermore, BCAAs play an important role in plant drought tolerance as an alternative source of respiratory substrate [22]. In *Arabidopsis* under severe drought stress [38], the expression of the branched chain aminotransferase gene (*BCAT2*), a BCAAs biosynthetic enzyme, was induced, consistent with the high accumulation of BCAAs. BCAAs also accumulated significantly in wheat under drought stress, suggesting that this is a common response to drought stress in plant species. However, the gene expression level of the *TaBCAT* enzyme in wheat in this study was not consistent with the accumulation of BCAAs [39]. Notably, the drought-tolerant TaPYLox wheat maintained lower BCAAs under drought stress (Fig 4a, 4c and 4e), suggesting that ABA-dependent drought-tolerant wheat might keep the content of these amino acids low. Alanine is also increased by drought stress, and this metabolite showed a similar trend to BCAAs between Null and TaPYLox (Fig 6, S3 and S6 Tables). It has been reported that drought stress promotes the synthesis of alanine from pyruvate. Therefore, these amino acids might be used as breeding markers for drought-tolerant wheat. Under drought stress, the shikimate metabolic pathway was inhibited and the levels of 3-dehydroshikimate and shikimate decreased (Figs 4 and 6). The shikimate pathway biosynthesizes aromatic amino acids such as L-tryptophan, L-phenylalanine and L-tyrosine, which are known to be precursors of many natural products involved in growth and disease resistance, such as auxins, alkaloids, phytoalexins, and cell wall components. Thus the shikimate pathway plays an important role in plant growth and environmental response [40]. In other words, it is conceivable that the reason why growth is suppressed by drought is that the production of active molecules associated with growth and disease resistance is reduced.

Organic acids such as cis-aconitate, fumarate and D-malate, which are TCA cycle intermediates, have been reported to increase in response to drought stress, whereas organic acids such as α-ketoglutarate and succinate decreased. Although the roles of organic acids in drought response and stress adaptation are not well understood, drought-induced disruption of the TCA cycle might lead to the accumulation of organic acids [24, 25, 41].

Flavonoids are synthesized through the phenylpropanoid pathway, transforming phenylalanine into 4-coumaroyl-CoA, which finally enters the flavonoid biosynthesis pathway [42]. The results (S3 Table) indicate that the amount of phenylalanine gradually increases with the severity of drought. This suggests that more phenylalanine is required to sustain plant life activities in response to harsh environmental conditions [43]. Similar, overexpression of glucosyltransferase UGT76E11 improves drought stress defense by elevating flavonoid content under drought conditions in Arabidopsis [44].

Upon perception of drought stress, plants accumulate ABA, which in turn triggers a series of physiological and metabolic changes aimed at conserving water and maintaining cellular homeostasis. We found that the 11 different metabolites of TaPYLox varied between ABA treatment and drought stress (S4b Fig and S5 Table). For instance, tagatose accumulated more in ABA-treated TaPYLox than in Null, and under drought stress conditions the compound accumulated in both Null and TaPYLox lines (Fig 3a). These results suggest that tagatose is a metabolite that increases in an ABA-dependent manner. Tagatose, which is generally known as a rare sugar, has been reported to accumulate more in drought-tolerant wheat lines than in normal wheat under drought stress conditions [45]. Interestingly, administration of tagatose to plants has also been reported to suppress plant pathogens [46]. Compared with raffinose and oligosaccharides, which are known to act as compatible solutes, tagatose has fewer reports in relation to drought stress, but it might play an important role in drought tolerance in wheat [38, 47].

Similarly, L-serine accumulated more in ABA-treated TaPYLox than in Null and, under drought stress conditions, accumulated in both Null and TaPYLox lines (Fig 3c). This suggests that L-serine is regulated in an ABA-dependent manner. The expression of the *SR1* gene, encoding the L-serine-inactivating enzyme, was reduced with ABA treatment in both Null and TaPYLox, and its expression level was further reduced in both Null and TaPYLox under drought stress conditions (S5a Fig). This suggests that the *SR1* gene is a key enzyme that determines the endogenous level of L-serine. It has also been reported that drought stress increases L-serine levels in wheat and other plant species [33, 48, 49]. L-serine is synthesized from 3-phosphoglycerate, an intermediate in the glycolytic pathway, and can then produce the osmoprotectant choline [50]. This relationship is consistent with the increase in L-serine under drought stress being a metabolic response of plants to adapt to drought stress. Moreover, ABA signaling pathways activate the expression of numerous drought-responsive genes, including those encoding for dehydrins, late embryogenesis abundant (LEA) proteins, and aquaporins, which collectively enhance the plant’s ability to withstand water-deficit conditions [51].

In conclusion, comprehensive metabolic profiling revealed changes in drought stress-induced ABA-dependent metabolites and ABA-independent drought stress-responsive metabolites. The ABA-dependent molecular markers revealed in TaPYLox wheat can be used not only for the selection of drought-tolerant lines from various wheat genotypes with different traits, but also as a diagnostic tool in wheat cultivation. The knowledge gained from this study will enable the development of new breeding methods, such as chemical analysis breeding, to supplement conventional and molecular breeding based on genetic information.

## Supporting information

S1 Fig Morphological and temperature changes under drought stress treatment over time. (a) Water supply was stopped from the 31st day after transplantation, and the amount of water loss was measured on days 0, 2, 4, and 6. WW denotes well-watered, DC denotes drought condition. Mean and standard deviation of three repetitions. Different letters indicate significant differences (Tukey–Kramer test, P < 0.05). (b) Morphological photographs and thermal images of plants under well-watered (WW) and drought stress (DC) conditions over time from 31 d after transplantation. The number of repetitions was three pots per treatment; representative images are shown.
(PDF)

S2 Fig Metabolites identified by GC–MS. (a) Data from GC–MS experiments were processed by Unknowns Analysis (Agilent Technologies) to identify metabolite quantities. The compound identification library used was the Agilent Fiehn library. Process denotes days of drought treatment (0 to 6 d); Detection denotes the total number of metabolites detected; Constant denotes the number of metabolites identified in common across different treatments. (b) A hierarchical cluster tree was created using MPP to collect and visualize compounds with similar patterns of variation. Red color indicates high accumulation, yellow an intermediate accumulation, and blue a low accumulation. (c) Principal component analysis was used to examine the interrelated effects of WW and DC conditions over time on metabolic profiles.
(PDF)

S3 Fig Metabolites altered by drought stress. Metabolite contents under drought condition (DC2, 4, and 6, sampled at 2, 4 and 6 d of drought treatment, respectively) were analyzed in comparison with well-watered plants (WW). The number of compounds in the overlaps between DC and WW comparison groups with significantly increased or decreased contents are shown in the Venn diagrams. The results analyzed by Unknowns Analysis were converted to.cef files for all identified and unidentified compounds and analyzed by MPP. Volcano Plot was performed to visualize the fold change and t-test results simultaneously. Paired t-test with P-value ≤ 0.05, fold change ≥ 1.25, and false discovery rate using Benjamini & Hochberg method. Refer to S2–S4 Tables for details of the compounds.
(PDF)

S4 Fig Metabolites varied with ABA treatment and drought condition in control line (Null) and TaPYLox. The Venn diagrams show the number of compounds with significantly increased (up) or decreased (down) contents in the comparison groups under ABA, well-watered (WW) or drought (DC) treatments. (a) Control line (Null). (b) TaPYLox. (c) Metabolites in common between Null and TaPYLox. The results analyzed by Unknowns Analysis were converted to.cef files for all identified and unidentified compounds and analyzed by MPP. Paired t -test with P-value ≤ 0.05, fold change ≥ 1.25, and false discovery rate with Benjamini & Hochberg method. Refer to S5 and S6 Tables for details of the compounds.
(PDF)

S5 Fig ABA-dependent decrease in L-serine inactivating gene expression. (a) Expression of *SR1* gene relative to *TaActin* (wheat housekeeping gene) in control line (Null) and TaPYLox (8–5 and 17–2) under well-watered condition (WW), ABA treatment (ABA) and drought condition (DC). (b) Gene expression in Null under drought stress treatment over time (days 0, 2, 4, 6). Mean and standard error of four repetitions. Different letters indicate significant differences (Tukey–Kramer test, P < 0.05).
(PDF)

S6 Fig Expression analysis of BCAAs metabolizing enzyme gene and 3-dehydroshikimic acid biosynthesis gene. Expression of *TaBCAT* gene (a) and *EMB3004* gene (c) relative to *TaActin* in control (Null) and TaPYLox lines (8–5 and 17–2) under well-watered condition (WW), ABA treatment (ABA) and drought condition (DC). (b) and (d), Gene expression changes in Null in drought stress treatment over time (days 0, 2, 4, 6). Mean and standard error of four of repetitions. Different letters indicate significant differences (Tukey–Kramer test, P < 0.05).
(PDF)

S7 Fig Analysis of LEA gene expression increased by drought stress. (a) expression of *LEA* gene relative to *TaActin* in control (Null) and TaPYLox lines (8–5 and 17–2) under well-watered condition (WW), ABA treatment (ABA) and drought condition (DC). (b) gene expression in Null under drought stress treatment over time (days 0, 2, 4, 6). Mean and standard error of four repetitions. Different letters indicate significant differences (Tukey–Kramer test, P < 0.05).
(PDF)

S1 Table Primer sets used in real-time PCR. *TaActin* is a wheat housekeeping gene; *SR1* is an L-serine inactivating gene; *TaBCAT* is a BCAAs metabolizing enzyme gene; *EMB3004* is a 3-dehydroshikimic acid biosynthesis gene; *LEA* is an ABA-responsive gene.
(XLSX)

S2 Table The compounds identified in each sample under drought stress treatment over time. The S2 Table provides the raw data for S2a Fig. Data from GC–MS experiments were processed using Unknowns Analysis (Agilent Technologies) to identify metabolite quantities. The number of repetitions was six. Compounds were detected using the “Fiehn” library as the database. "Detection" denotes the total number of metabolites detected, while "Constant" refers to the number of metabolites identified in common across different treatments.
(XLSX)

S3 Table Various compounds and their relative contents detected by GC-MS under drought stress treatment over time. The results, analyzed by Unknowns Analysis for all identified compounds, were converted to.cef files and further analyzed using MPP.
(XLSX)

S4 Table Metabolites increased and decreased by drought stress. Metabolites accumulating after drought stress (DC2, 4, and 6, respectively) were compared with those under well-watered conditions (WW). Paired t-tests were performed with P ≤ 0.05, fold change ≥ 1.25, and false discovery rate by Benjamini & Hochberg method.
(XLSX)

S5 Table The number of detections and constants and the names of the specific compounds that were identified for each sample in TaPYLox. Data from GC–MS experiments were processed by Unknowns Analysis (Agilent Technologies) to identify metabolite quantities. The number of repetitions was six. Compounds were detected using the “Fiehn” library as the database. "Detection" denotes the total number of metabolites detected, while "Constant" refers to the number of metabolites identified in common across different treatments.
(XLSX)

S6 Table Various compounds and their relative contents detected by GC-MS in TaPYLox. The results, analyzed by Unknowns Analysis for all identified compounds, were converted to.cef files and further analyzed using MPP.
(XLSX)

S7 Table Metabolites varied by ABA treatment and drought condition (DC) in control line (Null) and TaPYLox. The results analyzed by Unknowns Analysis for all identified and unidentified compounds were converted to.cef files and analyzed by MPP. Paired t-tests were performed with P ≤ 0.05, fold change ≥ 1.25, and false discovery rate by Benjamini & Hochberg method.
(XLSX)

## References

[1] Godfray HCJ, Beddington JR, Crute IR, Haddad L, Lawrence D, Muir JF, et al. Food security: the challenge of feeding 9 billion people. Science. 2010;327: 812–818. doi: 10.1126/science.1185383 20110467

[2] Tilman D, Balzer C, Hill J, Befort BL. Global food demand and the sustainable intensification of agriculture. Proc Natl Acad Sci U S A. 2011;108: 20260–20264. doi: 10.1073/pnas.1116437108 22106295 PMC3250154

[3] Dasgupta Y, Golovine K, Nieborowska-Skorska M, Luo L, Matlawska-Wasowska K, Mullighan CG, et al. Drugging DNA repair to target T-ALL cells. Leuk Lymphoma. 2018;59: 1746–1749. doi: 10.1080/10428194.2017.1397662 29115896 PMC5940588

[4] Eckardt NA, Cominelli E, Galbiati M, Tonelli C. The future of science: food and water for life. Plant Cell. 2009;21: 368–372. doi: 10.1105/tpc.109.066209 19252079 PMC2660623

[5] Shiferaw B, Prasanna BM, Hellin J, Bänziger M. Crops that feed the world 6. Past successes and future challenges to the role played by maize in global food security. Food Sec. 2011;3: 307–327. doi: 10.1007/s12571-011-0140-5

[6] Abdelrahman M, Burritt DJ, Gupta A, Tsujimoto H, Tran L-SP. Heat stress effects on source-sink relationships and metabolome dynamics in wheat. J Exp Bot. 2020;71: 543–554. doi: 10.1093/jxb/erz296 31232445

[7] Zhao C, Liu B, Piao S, Wang X, Lobell DB, Huang Y, et al. Temperature increase reduces global yields of major crops in four independent estimates. Proc Natl Acad Sci U S A. 2017;114: 9326–9331. doi: 10.1073/pnas.1701762114 28811375 PMC5584412

[8] Xiong L, Schumaker KS, Zhu J-K. Cell signaling during cold, drought, and salt stress. Plant Cell. 2002;14 Suppl: S165–183. doi: 10.1105/tpc.000596 12045276 PMC151254

[9] Zhu J-K. Abiotic Stress Signaling and Responses in Plants. Cell. 2016;167: 313–324. doi: 10.1016/j.cell.2016.08.029 27716505 PMC5104190

[10] Cutler SR, Rodriguez PL, Finkelstein RR, Abrams SR. Abscisic acid: emergence of a core signaling network. Annu Rev Plant Biol. 2010;61: 651–679. doi: 10.1146/annurev-arplant-042809-112122 20192755

[11] Fujii H, Chinnusamy V, Rodrigues A, Rubio S, Antoni R, Park S-Y, et al. In vitro reconstitution of an abscisic acid signalling pathway. Nature. 2009;462: 660–664. doi: 10.1038/nature08599 19924127 PMC2803041

[12] Ma Y, Szostkiewicz I, Korte A, Moes D, Yang Y, Christmann A, et al. Regulators of PP2C phosphatase activity function as abscisic acid sensors. Science. 2009;324: 1064–1068. doi: 10.1126/science.1172408 19407143

[13] Park S-Y, Fung P, Nishimura N, Jensen DR, Fujii H, Zhao Y, et al. Abscisic acid inhibits type 2C protein phosphatases via the PYR/PYL family of START proteins. Science. 2009;324: 1068–1071. doi: 10.1126/science.1173041 19407142 PMC2827199

[14] Shinozaki K, Yamaguchi-Shinozaki K. Molecular responses to dehydration and low temperature: differences and cross-talk between two stress signaling pathways. Curr Opin Plant Biol. 2000;3: 217–223. 10837265

[15] Uno Y, Furihata T, Abe H, Yoshida R, Shinozaki K, Yamaguchi-Shinozaki K. Arabidopsis basic leucine zipper transcription factors involved in an abscisic acid-dependent signal transduction pathway under drought and high-salinity conditions. Proc Natl Acad Sci U S A. 2000;97: 11632–11637. doi: 10.1073/pnas.190309197 11005831 PMC17252

[16] Fujita Y, Nakashima K, Yoshida T, Katagiri T, Kidokoro S, Kanamori N, et al. Three SnRK2 protein kinases are the main positive regulators of abscisic acid signaling in response to water stress in Arabidopsis. Plant Cell Physiol. 2009;50: 2123–2132. doi: 10.1093/pcp/pcp147 19880399

[17] Fujita Y, Yoshida T, Yamaguchi-Shinozaki K. Pivotal role of the AREB/ABF-SnRK2 pathway in ABRE-mediated transcription in response to osmotic stress in plants. Physiol Plant. 2013;147: 15–27. doi: 10.1111/j.1399-3054.2012.01635.x 22519646

[18] Sakuma Y, Maruyama K, Osakabe Y, Qin F, Seki M, Shinozaki K, et al. Functional analysis of an Arabidopsis transcription factor, DREB2A, involved in drought-responsive gene expression. Plant Cell. 2006;18: 1292–1309. doi: 10.1105/tpc.105.035881 16617101 PMC1456870

[19] Tran L-SP, Nakashima K, Sakuma Y, Simpson SD, Fujita Y, Maruyama K, et al. Isolation and functional analysis of Arabidopsis stress-inducible NAC transcription factors that bind to a drought-responsive cis-element in the early responsive to dehydration stress 1 promoter. Plant Cell. 2004;16: 2481–2498. doi: 10.1105/tpc.104.022699 15319476 PMC520947

[20] Abe H, Yamaguchi-Shinozaki K, Urao T, Iwasaki T, Hosokawa D, Shinozaki K. Role of arabidopsis MYC and MYB homologs in drought- and abscisic acid-regulated gene expression. Plant Cell. 1997;9: 1859–1868. doi: 10.1105/tpc.9.10.1859 9368419 PMC157027

[21] Arbona V, Manzi M, Ollas C de, Gómez-Cadenas A. Metabolomics as a tool to investigate abiotic stress tolerance in plants. Int J Mol Sci. 2013;14: 4885–4911. doi: 10.3390/ijms14034885 23455464 PMC3634444

[22] Pires MV, Pereira AA Júnior, Medeiros DB, Daloso DM, Pham PA, Barros KA, et al. The influence of alternative pathways of respiration that utilize branched-chain amino acids following water shortage in Arabidopsis. Plant Cell Environ. 2016;39: 1304–1319. doi: 10.1111/pce.12682 26616144

[23] Nakabayashi R, Mori T, Saito K. Alternation of flavonoid accumulation under drought stress in Arabidopsis thaliana. Plant Signal Behav. 2014;9: e29518. doi: 10.4161/psb.29518 25763629 PMC4203635

[24] Bowne JB, Erwin TA, Juttner J, Schnurbusch T, Langridge P, Bacic A, et al. Drought responses of leaf tissues from wheat cultivars of differing drought tolerance at the metabolite level. Mol Plant. 2012;5: 418–429. doi: 10.1093/mp/ssr114 22207720

[25] Gregorová Z, Kováčik J, Klejdus B, Maglovski M, Kuna R, Hauptvogel P, et al. Drought-Induced Responses of Physiology, Metabolites, and PR Proteins in Triticum aestivum. J Agric Food Chem. 2015;63: 8125–8133. doi: 10.1021/acs.jafc.5b02951 26330002

[26] Wu D, Shen Q, Cai S, Chen Z-H, Dai F, Zhang G. Ionomic responses and correlations between elements and metabolites under salt stress in wild and cultivated barley. Plant Cell Physiol. 2013;54: 1976–1988. doi: 10.1093/pcp/pct134 24058150

[27] Kobayashi F, Takumi S, Nakata M, Ohno R, Nakamura T, Nakamura C. Comparative study of the expression profiles of the Cor/Lea gene family in two wheat cultivars with contrasting levels of freezing tolerance. Physiol Plant. 2004;120: 585–594. doi: 10.1111/j.0031-9317.2004.0293.x 15032820

[28] Allwood JW, Chandra S, Xu Y, Dunn WB, Correa E, Hopkins L, et al. Profiling of spatial metabolite distributions in wheat leaves under normal and nitrate limiting conditions. Phytochemistry. 2015;115: 99–111. doi: 10.1016/j.phytochem.2015.01.007 25680480 PMC4518043

[29] Mega R, Abe F, Kim J-S, Tsuboi Y, Tanaka K, Kobayashi H, et al. Tuning water-use efficiency and drought tolerance in wheat using abscisic acid receptors. Nat Plants. 2019;5: 153–159. doi: 10.1038/s41477-019-0361-8 30737511

[30] Huang T, Jander G. Abscisic acid-regulated protein degradation causes osmotic stress-induced accumulation of branched-chain amino acids in Arabidopsis thaliana. Planta. 2017;246: 737–747. doi: 10.1007/s00425-017-2727-3 28668976

[31] Batista-Silva W, Heinemann B, Rugen N, Nunes-Nesi A, Araújo WL, Braun H-P, et al. The role of amino acid metabolism during abiotic stress release. Plant Cell Environ. 2019;42: 1630–1644. doi: 10.1111/pce.13518 30632176

[32] Lanzinger A, Frank T, Reichenberger G, Herz M, Engel K-H. Metabolite profiling of barley grain subjected to induced drought stress: responses of free amino acids in differently adapted cultivars. J Agric Food Chem. 2015;63: 4252–4261. doi: 10.1021/acs.jafc.5b01114 25867895

[33] Ullah N, Yüce M, Neslihan Öztürk Gökçe Z, Budak H. Comparative metabolite profiling of drought stress in roots and leaves of seven Triticeae species. BMC Genomics. 2017;18: 969. doi: 10.1186/s12864-017-4321-2 29246190 PMC5731210

[34] Kosová K, Vítámvás P, Prášil IT. Wheat and barley dehydrins under cold, drought, and salinity–what can LEA-II proteins tell us about plant stress response? Front Plant Sci. 2014;5: 343. doi: 10.3389/fpls.2014.00343 25071816 PMC4089117

[35] Itam M, Mega R, Tadano S, Abdelrahman M, Matsunaga S, Yamasaki Y, et al. Metabolic and physiological responses to progressive drought stress in bread wheat. Sci Rep. 2020;10: 17189. doi: 10.1038/s41598-020-74303-6 33057205 PMC7560863

[36] Taylor NL, Heazlewood JL, Day DA, Millar AH. Lipoic Acid-Dependent Oxidative Catabolism of α-Keto Acids in Mitochondria Provides Evidence for Branched-Chain Amino Acid Catabolism in Arabidopsis. Plant Physiology. 2004;134: 838–848. doi: 10.1104/pp.103.035675 14764908 PMC344558

[37] Araújo WL, Ishizaki K, Nunes-Nesi A, Larson TR, Tohge T, Krahnert I, et al. Identification of the 2-hydroxyglutarate and isovaleryl-CoA dehydrogenases as alternative electron donors linking lysine catabolism to the electron transport chain of Arabidopsis mitochondria. Plant Cell. 2010;22: 1549–1563. doi: 10.1105/tpc.110.075630 20501910 PMC2899879

[38] Urano K, Maruyama K, Ogata Y, Morishita Y, Takeda M, Sakurai N, et al. Characterization of the ABA-regulated global responses to dehydration in Arabidopsis by metabolomics. Plant J. 2009;57: 1065–1078. doi: 10.1111/j.1365-313X.2008.03748.x 19036030

[39] Buffagni V, Vurro F, Janni M, Gullì M, Keller AA, Marmiroli N. Shaping Durum Wheat for the Future: Gene Expression Analyses and Metabolites Profiling Support the Contribution of BCAT Genes to Drought Stress Response. Front Plant Sci. 2020;11: 891. doi: 10.3389/fpls.2020.00891 32719694 PMC7350509

[40] Maeda H, Dudareva N. The shikimate pathway and aromatic amino Acid biosynthesis in plants. Annu Rev Plant Biol. 2012;63: 73–105. doi: 10.1146/annurev-arplant-042811-105439 22554242

[41] Michaletti A, Naghavi MR, Toorchi M, Zolla L, Rinalducci S. Metabolomics and proteomics reveal drought-stress responses of leaf tissues from spring-wheat. Sci Rep. 2018;8: 5710. doi: 10.1038/s41598-018-24012-y 29632386 PMC5890255

[42] Falcone Ferreyra ML, Rius SP, Casati P. Flavonoids: biosynthesis, biological functions, and biotechnological applications. Front Plant Sci. 2012;3: 222. doi: 10.3389/fpls.2012.00222 23060891 PMC3460232

[43] Ramzan T, Shahbaz M, Maqsood MF, Zulfiqar U, Saman RU, Lili N, et al. Phenylalanine supply alleviates the drought stress in mustard (Brassica campestris) by modulating plant growth, photosynthesis, and antioxidant defense system. Plant Physiol Biochem. 2023;201: 107828. doi: 10.1016/j.plaphy.2023.107828 37329687

[44] Li Q, Yu H-M, Meng X-F, Lin J-S, Li Y-J, Hou B-K. Ectopic expression of glycosyltransferase UGT76E11 increases flavonoid accumulation and enhances abiotic stress tolerance in Arabidopsis. Plant Biol (Stuttg). 2018;20: 10–19. doi: 10.1111/plb.12627 28902451

[45] Guo R, Shi L, Jiao Y, Li M, Zhong X, Gu F, et al. Metabolic responses to drought stress in the tissues of drought-tolerant and drought-sensitive wheat genotype seedlings. AoB Plants. 2018;10: ply016. doi: 10.1093/aobpla/ply016 29623182 PMC5881611

[46] Mochizuki S, Fukumoto T, Ohara T, Ohtani K, Yoshihara A, Shigematsu Y, et al. The rare sugar D-tagatose protects plants from downy mildews and is a safe fungicidal agrochemical. Commun Biol. 2020;3: 423. doi: 10.1038/s42003-020-01133-7 32759958 PMC7406649

[47] Taji T, Ohsumi C, Iuchi S, Seki M, Kasuga M, Kobayashi M, et al. Important roles of drought- and cold-inducible genes for galactinol synthase in stress tolerance in Arabidopsis thaliana. Plant J. 2002;29: 417–426. doi: 10.1046/j.0960-7412.2001.01227.x 11846875

[48] The abundance of certain metabolites responds to drought stress in the highly drought tolerant plant Caragana korshinskii | Acta Physiologiae Plantarum. [cited 26 Jun 2024]. https://link.springer.com/article/10.1007/s11738-017-2412-y

[49] Duque P. A role for SR proteins in plant stress responses. Plant Signal Behav. 2011;6: 49–54. doi: 10.4161/psb.6.1.14063 21258207 PMC3122005

[50] Metabolite Adjustments in Drought Tolerant and Sensitive Soybean Genotypes in Response to Water Stress | PLOS ONE. [cited 26 Jun 2024]. https://journals.plos.org/plosone/article?id=10.1371/journal.pone.0038554 22685583 10.1371/journal.pone.0038554 PMC3369847

[51] Shinozaki K, Yamaguchi-Shinozaki K. Functional genomics in plant abiotic stress responses and tolerance: From gene discovery to complex regulatory networks and their application in breeding. Proceedings of the Japan Academy, Series B. 2022;98: 470–492. doi: 10.2183/pjab.98.024 36216536 PMC9614206

