# Supplementary material for: Metabolic profiles in drought-tolerant wheat with enhanced abscisic acid sensitivity
Source: PLoS One. 2024 Jul 22;19(7):e0307393. doi: 10.1371/journal.pone.0307393 (PMC11262632; doi:10.1371/journal.pone.0307393)
Supplement: S1 Fig — (a) Water supply was stopped from the 31st day after transplantation, and the amount of water loss was measured on days 0, 2, 4, and 6. WW denotes well-watered, DC denotes drought condition. Mean and standard deviation of three repetitions. Different letters indicate significant differences (Tukey–Kramer test, P < 0.05). (b) Morphological photographs and thermal images of plants under well-watered (WW) and drought stress (DC) conditions over time from 31 d after transplantation. The number of repetitions was three pots per treatment; representative images are shown. (PDF) [file pone.0307393.s001.pdf]

(a)

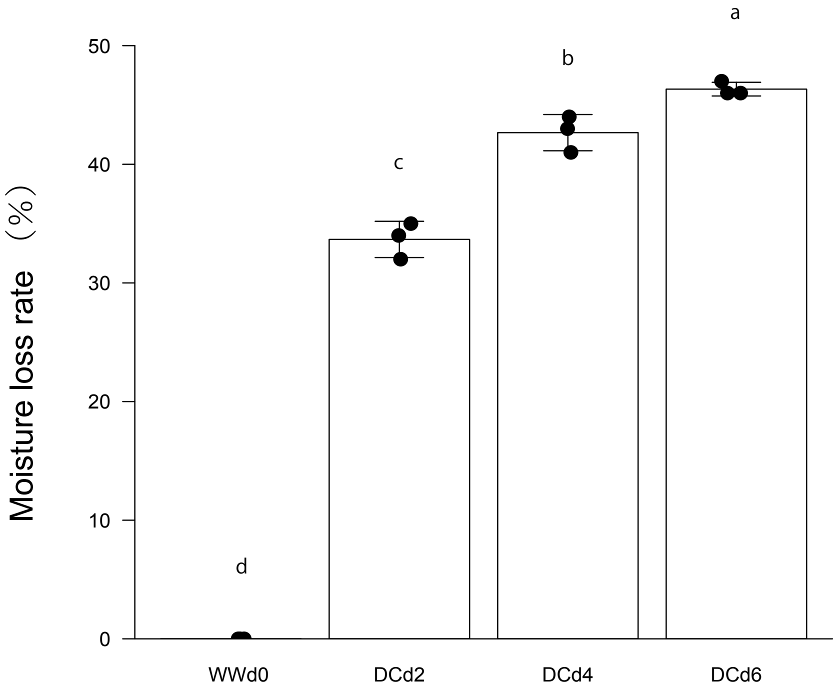

(b)

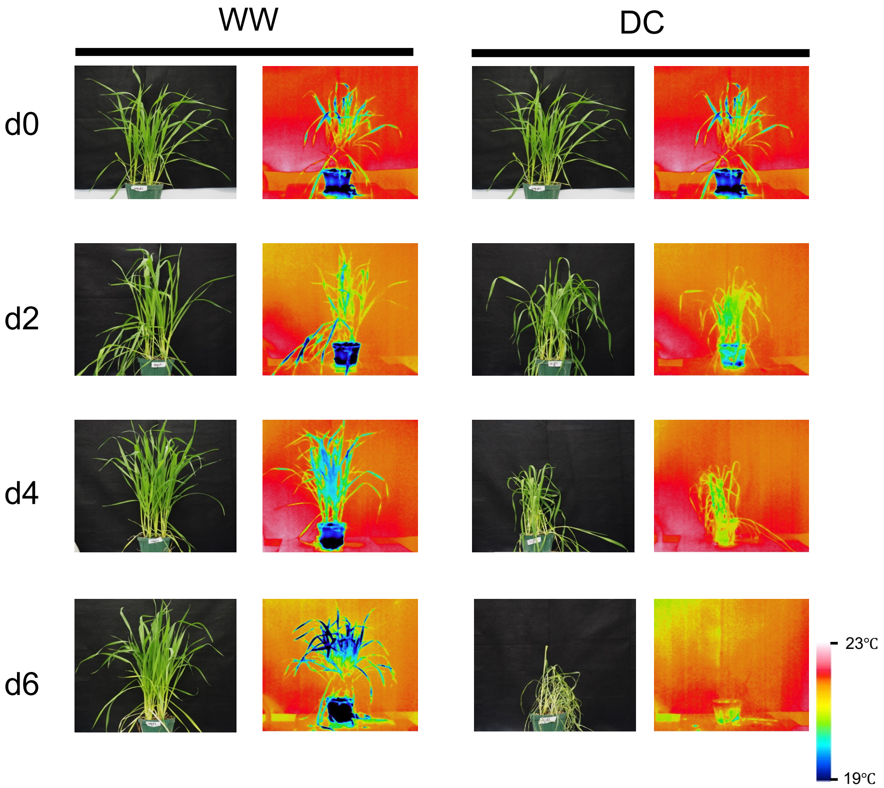

S1 Fig. Morphological changes and temperature changes due to drought stress treatment over time.
