# Supplementary material for: Metabolic profiles in drought-tolerant wheat with enhanced abscisic acid sensitivity
Source: PLoS One. 2024 Jul 22;19(7):e0307393. doi: 10.1371/journal.pone.0307393 (PMC11262632; doi:10.1371/journal.pone.0307393)
Supplement: S2 Fig — (a) Data from GC–MS experiments were processed by Unknowns Analysis (Agilent Technologies) to identify metabolite quantities. The compound identification library used was the Agilent Fiehn library. Process denotes days of drought treatment (0 to 6 d); Detection denotes the total number of metabolites detected; Constant denotes the number of metabolites identified in common across different treatments. (b) A hierarchical cluster tree was created using MPP to collect and visualize compounds with similar patterns of variation. Red color indicates high accumulation, yellow an intermediate accumulation, and blue a low accumulation. (c) Principal component analysis was used to examine the interrelated effects of WW and DC conditions over time on metabolic profiles. (PDF) [file pone.0307393.s002.pdf]

(a)

Well-Watered (WW)

Process	Detection	Constant
d0	340	122
d2	340	127
d4	355	122
d6	364	128

Drought-Condition (DC)

Process	Detection	Constant
d0	340	122
d2	347	127
d4	369	134
d6	380	132

(b)

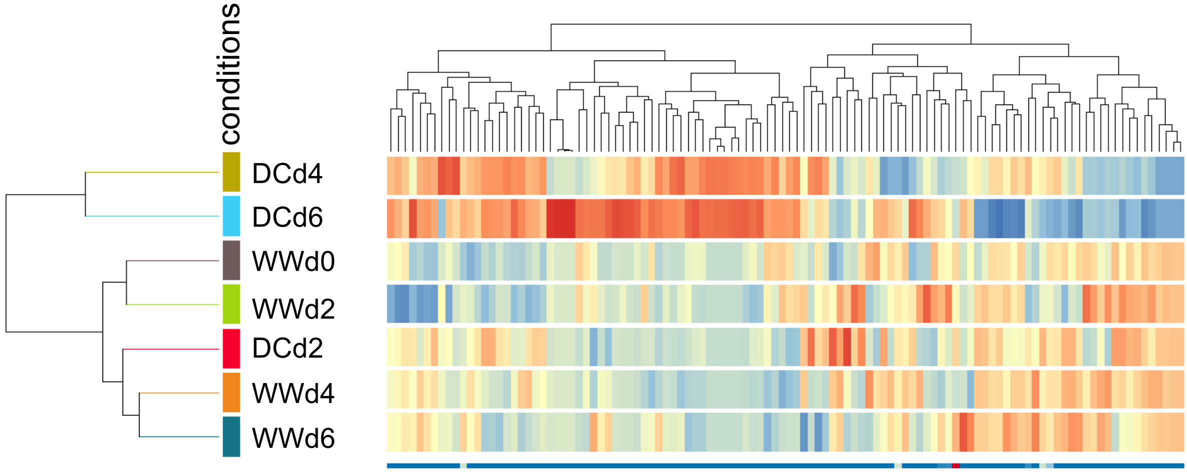

(c)

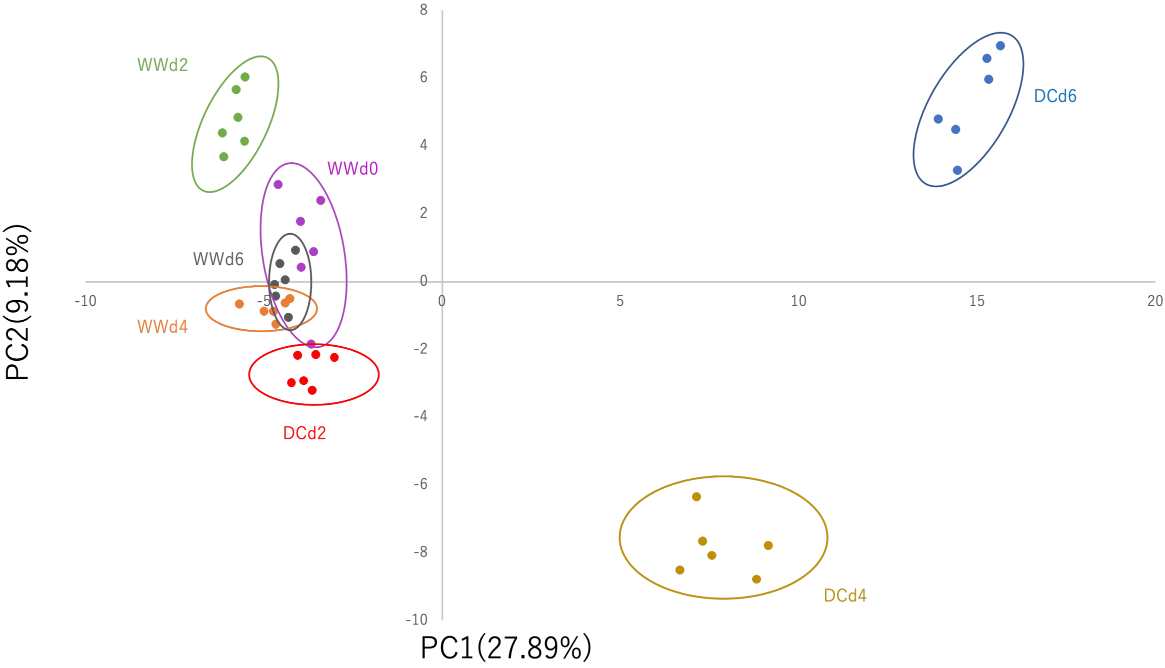

S2 Fig. Metabolites identified by GC-MS.
