# Supplementary material for: Metabolic profiles in drought-tolerant wheat with enhanced abscisic acid sensitivity
Source: PLoS One. 2024 Jul 22;19(7):e0307393. doi: 10.1371/journal.pone.0307393 (PMC11262632; doi:10.1371/journal.pone.0307393)
Supplement: S3 Fig — Metabolite contents under drought condition (DC2, 4, and 6, sampled at 2, 4 and 6 d of drought treatment, respectively) were analyzed in comparison with well-watered plants (WW). The number of compounds in the overlaps between DC and WW comparison groups with significantly increased or decreased contents are shown in the Venn diagrams. The results analyzed by Unknowns Analysis were converted to.cef files for all identified and unidentified compounds and analyzed by MPP. Volcano Plot was performed to visualize the fold change and t-test results simultaneously. Paired t-test with P-value ≤ 0.05, fold change ≥ 1.25, and false discovery rate using Benjamini & Hochberg method. Refer to S2–S4 Tables for details of the compounds. (PDF) [file pone.0307393.s003.pdf]

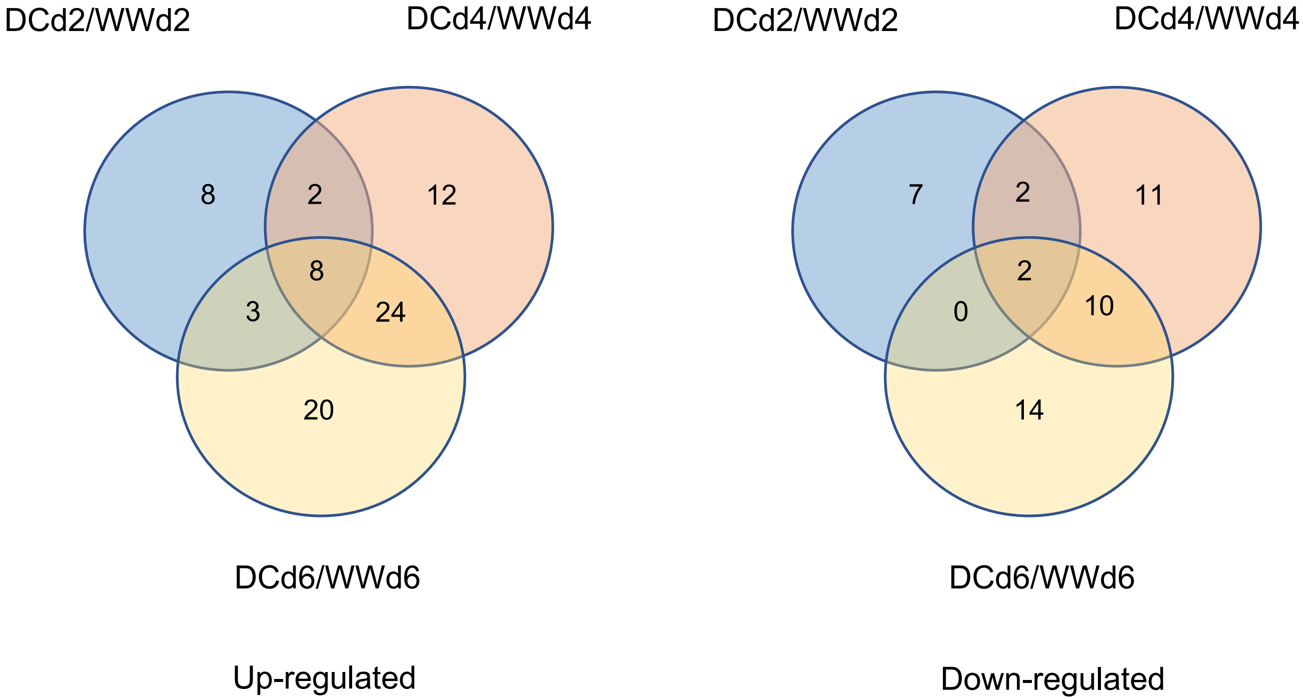

S3 Fig. Metabolite groups altered by drought stress.
