# Supplementary material for: Metabolic profiles in drought-tolerant wheat with enhanced abscisic acid sensitivity
Source: PLoS One. 2024 Jul 22;19(7):e0307393. doi: 10.1371/journal.pone.0307393 (PMC11262632; doi:10.1371/journal.pone.0307393)
Supplement: S4 Fig — The Venn diagrams show the number of compounds with significantly increased (up) or decreased (down) contents in the comparison groups under ABA, well-watered (WW) or drought (DC) treatments. (a) Control line (Null). (b) TaPYLox. (c) Metabolites in common between Null and TaPYLox. The results analyzed by Unknowns Analysis were converted to.cef files for all identified and unidentified compounds and analyzed by MPP. Paired t -test with P-value ≤ 0.05, fold change ≥ 1.25, and false discovery rate with Benjamini & Hochberg method. Refer to S5 and S6 Tables for details of the compounds. (PDF) [file pone.0307393.s004.pdf]

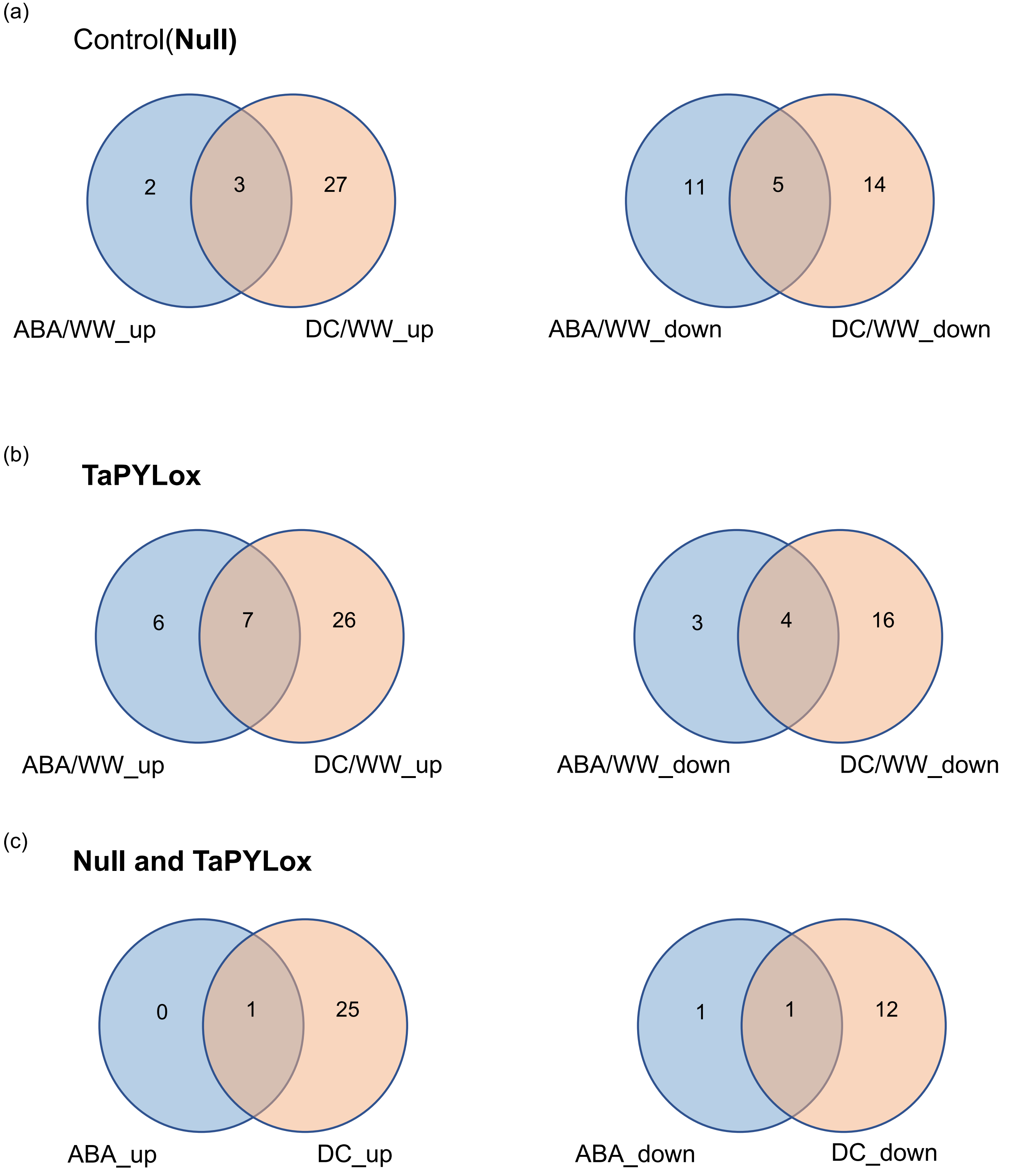

S4 Fig. Metabolites varied by ABA treatment and drought condition (DC) in control line (Null) and TaPYLox.
