# Supplementary figures and images for: Metabolic profiles in drought-tolerant wheat with enhanced abscisic acid sensitivity
Source: PLoS One. 2024 Jul 22;19(7):e0307393. doi: 10.1371/journal.pone.0307393 (PMC11262632; doi:10.1371/journal.pone.0307393)

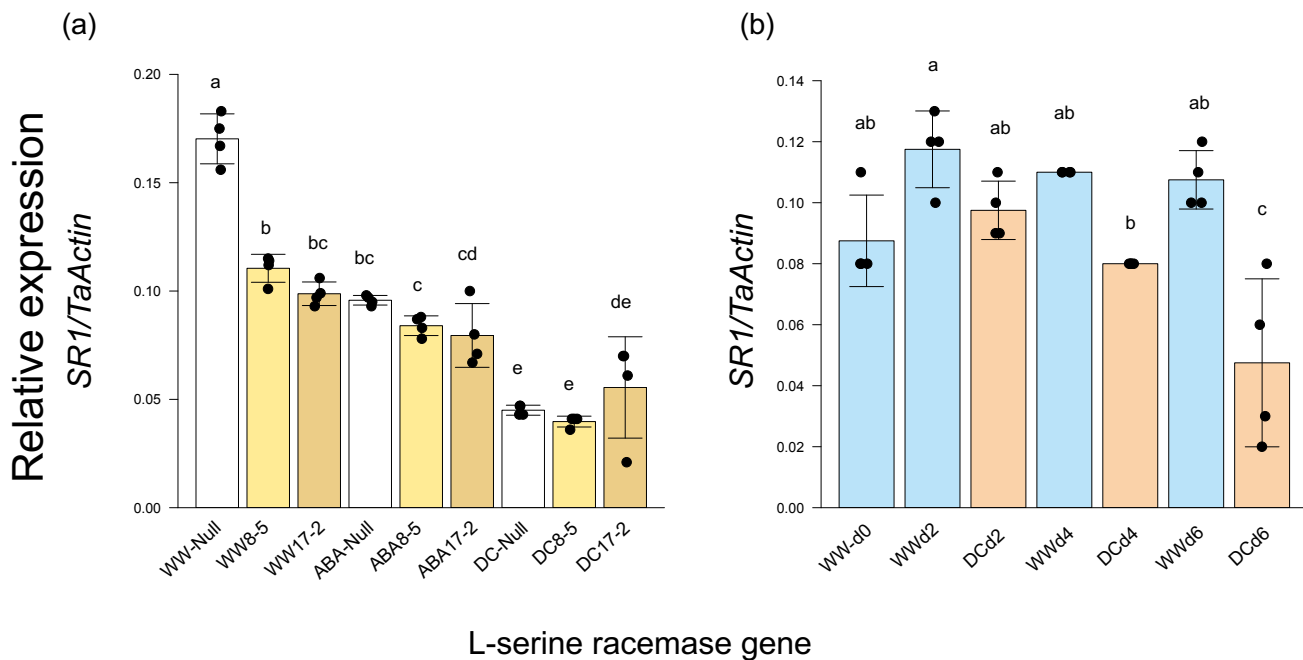

S5 Fig. ABA-dependent decrease in L-serine racemase gene expression analysis.

Supplement: S5 Fig — (a) Expression of SR1 gene relative to TaActin (wheat housekeeping gene) in control line (Null) and TaPYLox (8–5 and 17–2) under well-watered condition (WW), ABA treatment (ABA) and drought condition (DC). (b) Gene expression in Null under drought stress treatment over time (days 0, 2, 4, 6). Mean and standard error of four repetitions. Different letters indicate significant differences (Tukey–Kramer test, P < 0.05). (PDF) [file pone.0307393.s005.pdf]

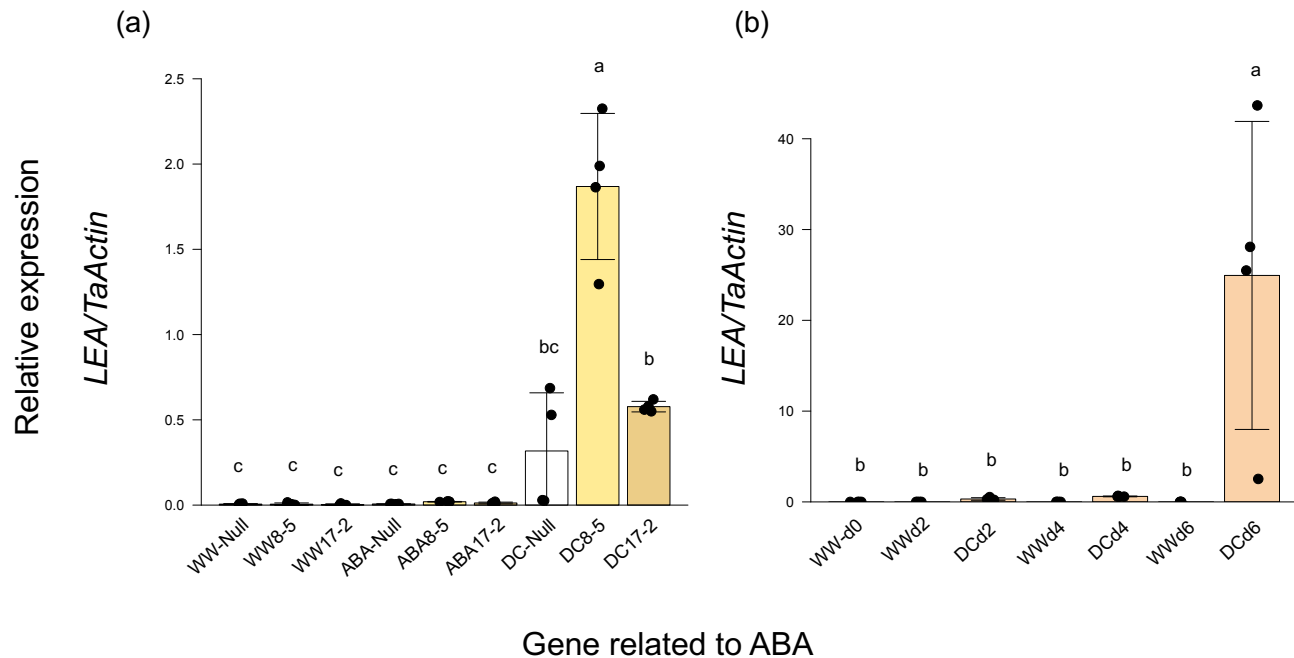

S7 Fig. Analysis of *LEA* gene expression increased by drought stress.

Supplement: S7 Fig — (a) expression of LEA gene relative to TaActin in control (Null) and TaPYLox lines (8–5 and 17–2) under well-watered condition (WW), ABA treatment (ABA) and drought condition (DC). (b) gene expression in Null under drought stress treatment over time (days 0, 2, 4, 6). Mean and standard error of four repetitions. Different letters indicate significant differences (Tukey–Kramer test, P < 0.05). (PDF) [file pone.0307393.s007.pdf]
