# Supplementary material for: Metabolic profiles in drought-tolerant wheat with enhanced abscisic acid sensitivity
Source: PLoS One. 2024 Jul 22;19(7):e0307393. doi: 10.1371/journal.pone.0307393 (PMC11262632; doi:10.1371/journal.pone.0307393)
Supplement: S6 Fig — Expression of TaBCAT gene (a) and EMB3004 gene (c) relative to TaActin in control (Null) and TaPYLox lines (8–5 and 17–2) under well-watered condition (WW), ABA treatment (ABA) and drought condition (DC). (b) and (d), Gene expression changes in Null in drought stress treatment over time (days 0, 2, 4, 6). Mean and standard error of four of repetitions. Different letters indicate significant differences (Tukey–Kramer test, P < 0.05). (PDF) [file pone.0307393.s006.pdf]

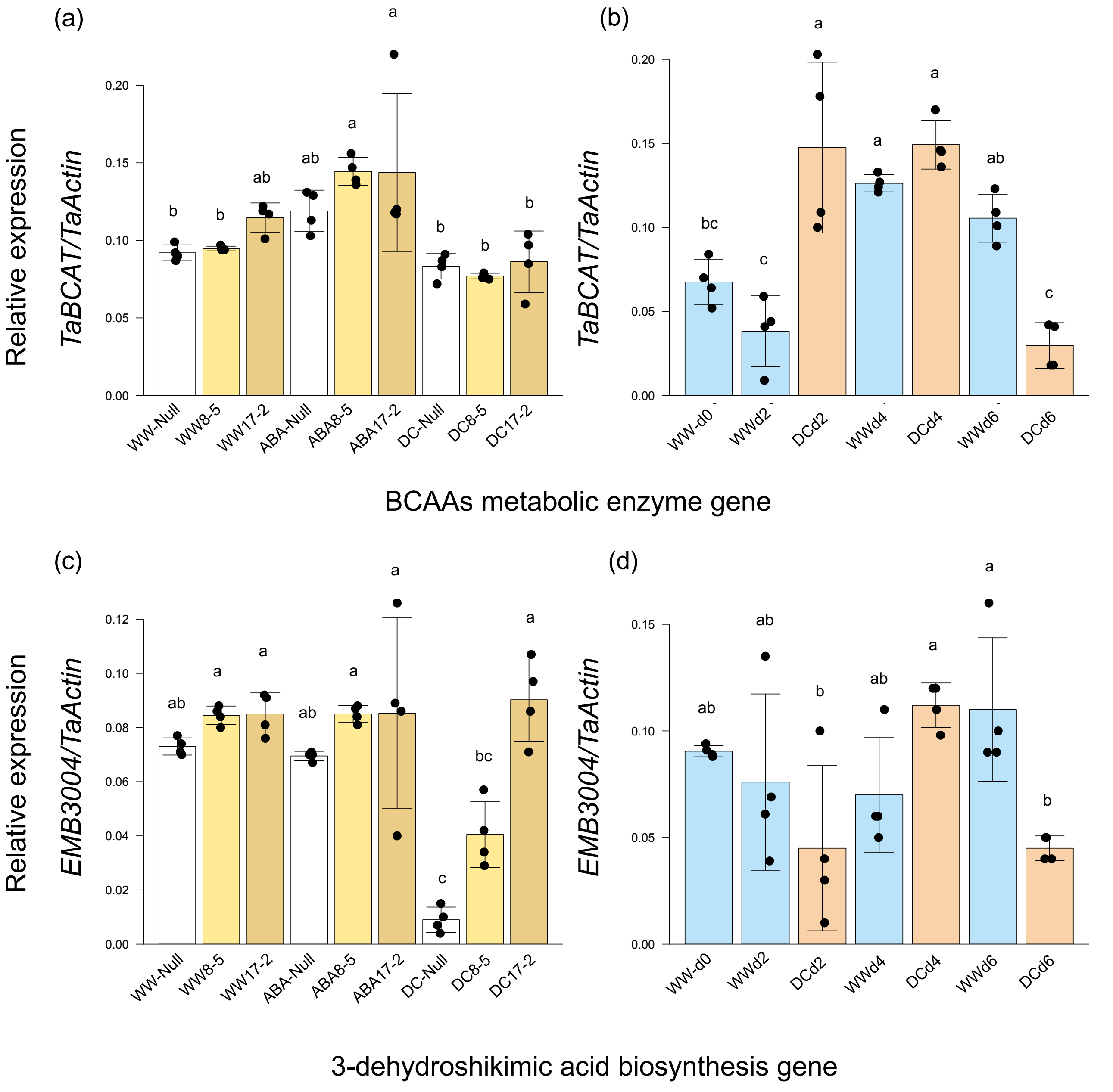

S6 Fig. Expression analysis of BCAAs metabolizing enzyme gene and 3-dehydroshikimic acid biosynthesis gene.
